# Effect of a Nutrition Intervention on Mediterranean Diet Adherence Among Firefighters: A Cluster Randomized Clinical Trial

**DOI:** 10.1001/jamanetworkopen.2023.29147

**Published:** 2023-08-17

**Authors:** Maria Soledad Hershey, Chia-Rui Chang, Mercedes Sotos-Prieto, Alejandro Fernandez-Montero, Sean B. Cash, Costas A. Christophi, Sara C. Folta, Carolyn Muegge, Vanessa Kleinschmidt, Steven Moffatt, Dariush Mozaffarian, Stefanos N. Kales

**Affiliations:** 1 Department of Environmental Health, Harvard T. H. Chan School of Public Health, Boston, Massachusetts; 2 Department of Biostatistics, Harvard T. H. Chan School of Public Health, Boston, Massachusetts; 3 Department of Preventive Medicine and Public Health, School of Medicine, Universidad Autónoma de Madrid, Madrid, Spain; 4 Centro de Investigación Biomédica en Red of Epidemiology and Public Health, Madrid, Spain; 5 Campus of International Excellence (CEI) Universidad Autónoma de Madrid (UAM), Spanish National Research Council (CSIC), Madrid, Spain; 6 Department of Occupational Medicine, University of Navarra, Pamplona, Spain; 7 Instituto de Investigación Sanitaria de Navarra (IdiSNA), Pamplona, Spain; 8 Friedman School of Nutrition Science and Policy, Tufts University, Boston, Massachusetts; 9 Cyprus International Institute for Environmental and Public Health, Cyprus University of Technology, Lemesos, Cyprus; 10 National Institute for Public Safety Health, Indianapolis, Indiana; 11 Occupational Medicine, Cambridge Hospital, Harvard Medical School, Cambridge, Massachusetts

## Abstract

**Importance:**

US firefighters are a working population at risk of chronic diseases, including obesity, cardiovascular disease, and cancer. This risk may be mitigated by a healthy diet.

**Objective:**

To evaluate the effect of a Mediterranean nutrition intervention using a behavioral/environmental approach (firefighter/fire station/home) at the individual participant level.

**Design, Setting, and Participants:**

This 12-month cluster randomized clinical trial included US career firefighters from fire stations and homes within 2 Indiana fire departments. Participants were randomized by fire station to either Mediterranean diet or control (usual care). The study was conducted from October 2016 to December 2019, and data were analyzed in November 2022.

**Intervention:**

For the first 12 months of the study, firefighters located at fire stations randomized to the intervention group were provided with access to supermarket discounts and free samples of Mediterranean diet foods, online nutrition education platforms, email announcements and reminders, family and peer education and support, and chef demonstrations. Firefighters in fire stations allocated to the control group received no intervention and were instructed to follow their usual diet.

**Main Outcomes and Measures:**

Change in dietary habits at 12 months as measured by a modified Mediterranean diet score (range, 0 to 51 points) at baseline and 6-month and 12-month follow-up. Cardiometabolic parameters were secondary outcomes.

**Results:**

Of 485 included firefighters, 458 (94.4%) were male, and the mean (SD) age was 47 (7.5) years. A total of 241 firefighters (27 fire stations) were randomized to the Mediterranean nutrition intervention, and 244 (25 fire stations) were randomized to usual diet. Outcomes were analyzed using generalized linear mixed models for modified Mediterranean diet score at 6 months (n = 336) and 12 months (n = 260), adjusting for baseline age, sex, race and ethnicity, fire department, physical activity, and waist circumference. In the intervention group compared with the control group, the modified Mediterranean diet score significantly increased by 2.01 points (95% CI, 0.62-3.40; *P* = .005) at 6 months and by 2.67 points (95% CI, 1.14-4.20; *P* = .001) at 12 months. Among secondary outcomes, changes in cardiometabolic risk factors were not statistically significant at 1 year. Results from analyses with multilevel multiple imputation for missingness were similar.

**Conclusions and Relevance:**

In this Mediterranean nutrition intervention of multicomponent behavioral/environmental changes, career firefighters had increased adherence to a Mediterranean diet.

**Trial Registration:**

ClinicalTrials.gov Identifier: NCT02941757

## Key Points

**Question:**

What is the effect of a workplace-based nutritional and behavioral intervention on adherence to Mediterranean diet principles at 12 months?

**Findings:**

In this cluster randomized clinical trial including 52 fire stations and 485 enrolled US career firefighters, the intervention group achieved significant improvements in Mediterranean diet adherence after a 12-month intervention.

**Meaning:**

The findings of this study support a multicomponent workplace intervention to change US career firefighters’ eating habits and lifestyle, suggesting further implementation in the US fire service as a chronic disease prevention strategy.

## Teaser

This cluster randomized clinical trial evaluates the effect of a workplace-based nutrition intervention on adherence to a Mediterranean diet among US career firefighters.

## Introduction

Efforts to improve the health of US firefighters are critical. The career fire service represents a working population at high risk of obesity, cardiovascular disease (CVD), and cancer.^1,2,3,4^ Despite hazards, including asphyxiation, burns, and other accidents, the most frequent cause of on-duty fatalities (44%) among US firefighters has been sudden cardiac death for nearly 3 decades (1977 to 2021).^5^ In the official *Fire Fighter Fatality Investigation and Prevention Program*,^6^ 9 of 31 recommendations aim to reduce fatalities from CVD, but few fire departments have adequate programs on nutrition, wellness, or fitness.

Behavioral efforts that focus on education and knowledge alone are often less effective than multicomponent interventions that incorporate changes to the environment. This is particularly relevant to career firefighters, whose lifestyles are highly influenced by the fire station culture and environment, where they typically spend 48 hours per week collectively eating, sleeping, and responding to emergencies.^7,8^ A systematic review encompassing 26 nutrition and physical activity assessments concluded that US firefighters have poor dietary habits, poor food environments, and low physical activity.^9^ Among different dietary patterns, a Mediterranean diet has the strongest evidence for benefits on CVD, adiposity, and related risk factors from long-term observational studies, controlled trials of risk factors, and randomized clinical trials of clinical events in high-risk populations.^10,11,12,13^ However, few studies among firefighters have addressed the role of a healthy diet in the development of noncommunicable diseases, and even fewer randomized clinical trials in firefighters have examined the efficacy of diet interventions on CVD risk factors.^9,14,15,16,17,18^ However, interest among firefighters is high; a cross-sectional survey demonstrated that US firefighters most often rated the Mediterranean diet as their favorite eating pattern (while being least receptive to strictly plant-only diets), and 75% wanted to learn more about healthy eating.^19^

The high prevalence of on-duty fatalities due to CVD, underlying chronic disease risk factors (eg, unhealthy diet, adiposity, hypertension, physical inactivity, occupational stress), and interest in healthier eating suggests US career firefighters may benefit from a Mediterranean nutrition intervention.^4,20,21^ The Mediterranean diet emphasizes minimally processed fiber-rich and bioactive-rich plant foods in an omnivorous diet that is highly diverse and palatable and does not require calorie counting nor prohibit any food but rather fosters variety and moderation.^22^ This report presents the primary findings of a 12-month workplace, cluster randomized clinical trial at the end of 1 year among US firefighters. The intervention included nutrition education, behavioral counseling, and environmental strategies to promote Mediterranean diet-adherence among Midwestern fire stations and firefighter homes.^1^

## Methods

### Study Design and Population

*Feeding America’s Bravest* was originally designed as a cluster randomized crossover trial conducted among career firefighters from the 52 fire stations of 2 Indiana Fire Departments (Indianapolis Fire Department, 45 stations; Fishers Fire Department, 7 stations). Due to attrition at follow-up visits, this article analyzes data from the first 12 months of the trial as a parallel cluster randomized clinical trial. Inclusion criteria included age of 18 years or older; regular assignment to a specific fire station; medical examination data within the last 2 years; and full, modified, or restricted duty or administrative staff status. Exclusion criteria included no fire department medical examination recorded in the last 2 years, age younger than 18 years, or inability to give informed consent.

Eligible participants were enrolled and cluster randomized by fire station at baseline to either a 12-month Mediterranean nutrition intervention (241 firefighters at 27 fire stations) or control (ie, usual diet; 244 firefighters at 25 fire stations). The 7 stations at Fishers Fire Department were invited to participate and subsequently randomized after the 45 stations in Indianapolis. Race and ethnicity data were self-reported via a questionnaire; participants indicated American Indian or Alaska Native, Asian, Black, Native Hawaiian or Other Pacific Islander, or White race and Hispanic or non-Hispanic ethnicity. Data were collected between November 2016 and July 2019. The study protocol was approved by the Harvard Institutional Review Board and is registered at ClinicalTrials.gov^23^; the study protocol can be found in Supplement 1. In accordance with the Declaration of Helsinki, all potential participants were informed of their right to refuse to participate or to withdraw from the study at any time without retribution. Written informed consent was obtained from all participants. More details on this study’s objective, design, and methods, including study sample size and allocation, have been published previously.^1^ This study followed the Consolidated Standards of Reporting Trials Extension (CONSORT Extension) reporting guideline for cluster randomized trials.^8^

### Intervention

From baseline through 12 months of follow-up, the Mediterranean nutrition intervention group was instructed to follow a Mediterranean diet using multicomponent nutrition intervention strategies. Education was provided via an online platform, which contained brochures on Mediterranean diet recommendations, a firefighter-specific Mediterranean diet pyramid, grocery shopping tips, sample recipes, video interviews with exemplary firefighters practicing the Mediterranean diet, and access to additional resources, such as chef-led cooking demonstrations in fire station kitchens. Additional behavioral and environmental approaches were adopted to promote the Mediterranean diet in the Mediterranean nutrition intervention group, including access to supermarket discounts, free samples of Mediterranean diet foods, email announcements and reminders, and family and peer education and support.^24^ The Mediterranean nutrition intervention goals were informed by Mediterranean diet recommendations,^25,26,27^ conversations with scientific and national fire service advisory boards, institutional review board–approved focus group findings in which firefighters and spouses identified key adaptations to the fire service for this study,^28^ as well as social cognitive theory principals involving education, participation, and incentives.^29^ These 17 goals consisted of the general recommendations for the food groups encouraged and discouraged by the Mediterranean diet (eMethods in Supplement 2). The control group received no intervention and were instructed to follow their usual diet without being provided any Mediterranean nutrition intervention study materials in the first 12 months.

### Outcome Assessment

Dietary information that most closely described participants’ general eating preferences and habits was obtained from a validated and previously used 13-domain (16-item) modified Mediterranean diet score (mMDS)^30^ administered at baseline and 6-month and 12-month study visits by the local study team.^30,31,32,33^ Dietary intake was also assessed using a validated 131-item semiquantitative food frequency questionnaire (FFQ) at baseline, which reflects the previous year’s habitual intake and has shown correlations against energy-adjusted nutrient intakes measured by diet records^34^ and plasma biomarkers of nutrients.^31^ A validation study in this population demonstrated that the 13-domain mMDS questionnaire was well correlated with the FFQ-derived mMDS (*r* = 0.74), with good concordance between questionnaires (κ = 0.76) for low vs high Mediterranean diet adherence.^30,31^

Information on self-reported sleep and physical activity were assessed with sleep^35^ and validated physical activity^33^ questionnaires administered within the online lifestyle questionnaire at each study visit. Sociodemographic characteristics, information on diet and lifestyle, and anthropometric measurements were collected in-person, while biochemical, fitness assessments, and medical history were collected through existing fire department fitness and medical records retrieved and matched to in-person study visits after informed consent was given.

#### Primary Outcome

The primary end point was the 12-month change in adherence to the Mediterranean diet, as measured by a validated 13-domain mMDS at baseline and the 6-month and 12-month study visits at the individual firefighter-level adjusting for clustering.^30,31^ mMDS components include the consumption of fast food, fruits, vegetables, desserts, fried food, fish, alcohol, wine, legumes, and nuts, as well as primary cooking oil, bread/starches, and beverages at home and work. Three domains are weighted by the proportion of meals eaten at the fire station and at home relative to the total number of meals per week, since meals at the workplace and at home may differ substantially. eTable 5 in Supplement 2 indicates each item’s scoring criteria and the derivation of the final mMDS for a total score range of 0 points (no adherence) to 51 points (maximum adherence).

#### Secondary Outcomes

Secondary outcomes included cardiometabolic parameters: body mass index (BMI), body fat percentage, waist circumference, plasma glucose, triglycerides, total cholesterol, high-density lipoprotein (HDL) cholesterol, and low-density lipoprotein (LDL) cholesterol. Height was measured with a standard clinic stadiometer and body weight with a calibrated scale while wearing light clothing with bare feet in private areas of the fire houses by trained public safety medial clinical staff at each study visit. Waist circumference was assessed using a tape measure fitted around each participant’s waist at the level of the iliac crest and measuring the circumference after expiration. A bioelectrical impedance analyzer (Tanita Corporation of America) was used to estimate body fat. Fasting blood samples were collected during fire department medical examinations, timed to the 6-month and 12-month endpoints (within 45 days). Plasma and serum samples were collected in the 15-mL ethylenediamine tetra-acetic acid tubes as appropriate for each assay, aliquoted, frozen at −80 °C, and then stored. Blood lipid profiles were determined using standardized automated high-throughput enzymatic analyses using cholesterol assay kit and reagents and triglyceride assay kit and reagents by ARCHITECT c System (Abbott Laboratories). LDL cholesterol was assessed using the Martin-Hopkins equation.

### Statistical Analysis

Baseline characteristics of each study group were summarized using descriptive statistics, with differences between groups assessed using a one-way analysis of variance for quantitative variables and χ^2^ test for qualitative variables. Baseline mMDS scores were derived from either the 13-domain mMDS (n = 374) or full FFQ (n = 110), and 6-month and 12-month scores were derived from the 13-domain mMDS. mMDS changes were compared within and between groups at baseline, 6, and 12 months using generalized linear mixed models with fire station–level and participant-level random intercepts to account for the cluster-randomized design and the longitudinal follow-up. Mean differences between the 2 arms were estimated for the mMDS score, mMDS components, and secondary end points using the mixed models while adjusting for age, sex, fire department, physical activity, race and ethnicity, and waist circumference.^1^ We corrected for multiple comparisons using the Simes multiple-test procedure. The intraclass correlation coefficient (ICC) was calculated between Mediterranean nutrition intervention and control group differences in mMDS changes, using Stata version 15.0 (StataCorp). *P* values for all analyses were 2-sided, with statistical testing based on a significance level less than .05.

Our prespecified primary analysis included all observations based on the intention-to-treat principle.^1^ We also performed multiple imputation analyses to address potential informative missingness due to loss to follow-up. Applying multilevel multiple imputation,^36,37^ we imputed time-varying variables with missingness to obtain 25 imputed data sets using the jomo package in R software version 4.1.0 (The R Foundation), except for the following baseline covariates: age, sex, race and ethnicity, education level, total daily energy intake, smoking status, chronic disease prevalence, and the proportion of meals consumed at the fire stations, which were imputed using the mean of observed values because these were not time-varying covariates.^38^ To test the robustness of the imputation model, influence analyses for the primary outcomes were performed using the leave-one-out method. Additionally, descriptive quality checks of the imputation models were assessed by histograms, kernel density plots, and summary statistics. Inference for multiple imputation analyses were conducted by pooling the results accounting for variability both within and between data sets.

## Results

### Baseline Characteristics

Among 485 US career firefighters enrolled in this trial, 458 (94.4%) were male, and the mean (SD) age was 47 (7.5) years. A total of 69 participants were Black (14.3%), 401 were White (82.9%), and 14 were another race or ethnicity (including American Indian or Alaska Native, Asian, Hispanic, or Native Hawaiian or Other Pacific Islander; 2.9%). After exclusion of 1 participant for total daily energy intake levels beyond predefined limits, 484 firefighters were included in the primary analysis (Figure). Most baseline characteristics were similar across study groups, including age, sex, body mass index, smoking status, marital status, education level, and self-reported chronic disease status (Table 1). Modest differences, by chance, were seen across study groups by race and ethnicity, waist circumference, and physical activity level, with the Mediterranean nutrition intervention group having a higher proportion of Black firefighters and sedentary firefighters and slightly lower waist circumference. These baseline characteristics were included in all models assessing the outcomes.

**Figure.**
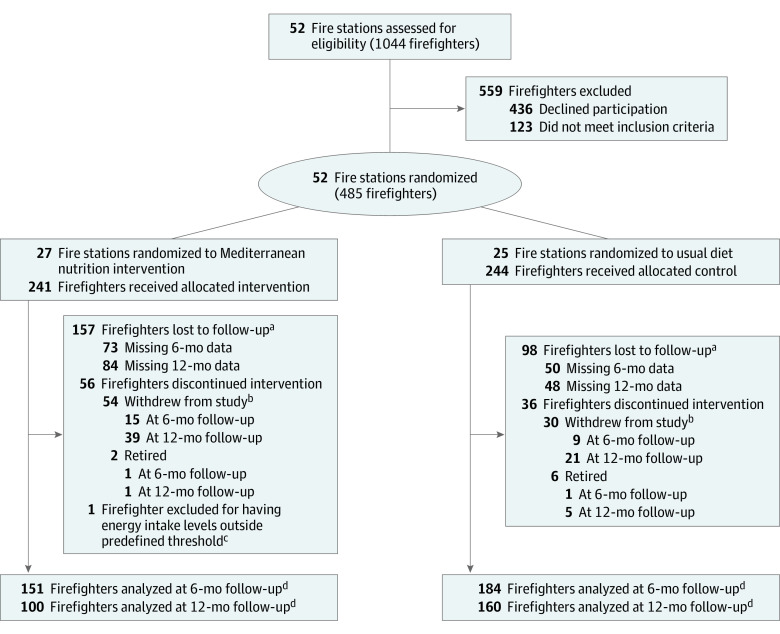
CONSORT Flow Diagram of Clusters and Individuals in the Feeding America’s Bravest Cluster Randomized Clinical Trial ^a^A total of 362 of 485 firefighters (74.6%) were retained. ^b^Reasons for withdrawal from study included no reason provided (n = 66), no longer wanted to participate because hadn’t been following diet (n = 5), no longer interested in study (n = 4), did not like the study intervention materials (n = 3), too busy to learn a new diet (n = 3), medical reason (n = 1), no longer wanted to complete study follow-up visits (n = 1), and felt diet was too difficult to follow (n = 1). ^c^Predefined total daily energy intake levels were defined as less than 8000 kcal/d. ^d^Our prespecified primary analysis included all observations based on the intention-to-treat principle.

**Table 1.** Baseline Characteristics of Feeding America’s Bravest Participants by Study Group

Characteristic	Total, No. (%)	
	Control	Mediterranean nutrition intervention
mMDS^a^		
Total, No.	244	240
Mean (SD)	23.41 (6.34)	22.87 (6.78)
Indianapolis Fire Department	657 (90)	621 (86)
Fishers Fire Department	75 (10)	99 (14)
Age		
Total, No.	239	231
Mean (SD), y	46.28 (7.74)	45.12 (8.53)
Sex		
Female	10 (4.1)	17 (7.1)
Male	234 (95.9)	223 (92.9)
Race and ethnicity		
Black	22 (9.0)	47 (19.6)
White	215 (88.1)	186 (77.5)
Other race or ethnicity^b^	7 (2.9)	7 (2.5)
Marital status		
Married	128 (84.2)	130 (76.9)
Never married, divorced, or widowed	24 (15.8)	39 (23.1)
BMI^c^		
Total, No.	236	234
Mean (SD)	30.14 (4.43)	29.92 (4.40)
Body fat percentage		
Total, No.	234	234
Mean (SD)	27.61 (6.38)	28.67 (6.64)
Waist circumference		
Total, No.	235	234
Mean (SD), cm	101.16 (12.48)	98.27 (12.48)
Glucose^d^		
Total, No.	240	233
Mean (SD), mg/dL	98.97 (18.99)	99.97 (19.85)
Total cholesterol^d^		
Total, No.	240	233
Mean (SD), mg/dL	195.08 (38.25)	198.44 (36.58)
HDL cholesterol		
Total, No.	240	233
Mean (SD), mg/dL	48.19 (11.51)	49.01 (11.17)
LDL cholesterol		
Total, No.	236	229
Mean (SD), mg/dL	122.05 (33.42)	124.36 (31.40)
Triglycerides^d^		
Total, No.	240	232
Mean (SD), mg/dL	125.99 (73.63)	124.65 (78.57)
Education level		
Bachelor’s degree or higher	51 (33.6)	56 (32.9)
Technical school/some college/associate degree	101 (66.5)	114 (67.1)
Total energy intake		
Total, No.	218	206
Mean (SD), kcal/d	2402 (1065)	2413 (1172)
Smoking status		
Current	13 (8.6)	17 (10.0)
Former	46 (30.3)	48 (28.2)
Never	93 (61.2)	105 (61.8)
Physical activity^e^		
No regular exercise	13 (8.8)	27 (16.3)
Regular modest exercise	40 (27.0)	27 (16.3)
Regular heavy exercise	95 (64.2)	112 (67.5)
Chronic conditions^f^		
Yes	77 (31.7)	67 (28.3)
No	166 (68.3)	170 (71.4)

Abbreviations: BMI, body mass index; HDL, high-density lipoprotein; LDL, low-density lipoprotein; mMDS, modified Mediterranean diet score.

SI conversion factor: To convert glucose to mmol/L, multiply by 0.0555; cholesterol to mmol/L, multiply by 0.0259; triglycerides to mmol/L, multiply by 0.0113.

^a^ mMDS ranged from 0 points (no adherence) to 51 points (maximum adherence).

^b^ The other race or ethnicity category included American Indian or Alaska Native, Asian, Hispanic, and Native Hawaiian or Other Pacific Islander. These categories were combined because of low numbers.

^c^ Calculated as weight in kilograms divided by height in meters squared.

^d^ Glucose and lipid profiles were determined using fasting blood samples collected during fire department medical examinations.

^e^ No regular exercise was defined as not participating regularly in programmed recreation, sport, or heavy physical activity and avoiding walking or exertion; regular modest exercise, participating regularly in recreation or work requiring modest physical activity, such as golf, horseback riding, calisthenics, gymnastics, table tennis, bowling, weightlifting, or yard work, walking for pleasure, and routinely using stairs; and regular heavy exercise, participating regularly in heavy physical exercise, such as running or jogging, swimming, cycling, rowing, skipping rope, running in place, or engaging in vigorous aerobic activity, such as tennis, basketball, or handball, for 10 to 60 minutes per week.

^f^ Defined as self-reported cardiovascular disease, hypertension, arrythmia, diabetes, dyslipidemia, or angina.

### Primary Outcome

The analyses included 244 firefighters from 25 fire stations in the control group and 240 firefighters from 27 fire stations in the Mediterranean nutrition intervention group (122 [25%] missing data). Participants with missing mMDS values had slightly lower baseline mMDS, greater allocation to the intervention group (140 of 240 [62.5%]), slightly higher body fat percentage and HDL cholesterol, and slightly lower total energy intake (eTable 1 in Supplement 2).

In the primary analyses, for the intervention group compared with the control group, the modified Mediterranean diet score significantly increased by 2.01 points (95% CI, 0.62-3.40; *P* = .005) at 6 months and by 2.67 points (95% CI, 1.14-4.20; *P* = .001) at 12 months (ICC = 0.03; 95% CI, 0.01-0.14) (Table 2). Evaluating components of the Mediterranean diet, the intervention group generally showed higher score improvements than the control group in most of the Mediterranean diet food groups, although most of these were not statistically significant except for increased points in cooking oil and breads/starches consumed at work, fish consumption, nut consumption, and a decrease in fast food and alcoholic beverages consumed (Table 3).

**Table 2.** Between-Group Differences for 6-Month and 12-Month Changes in Modified Mediterranean Diet Score (mMDS) by Study Group

Group	Mean (SD)			6-mo Follow-up, mean points (95% CI)^a^		12-mo Follow-up, mean points (95% CI)^a^	
	Baseline	6 mo	12 mo	6-mo Change	Between-group difference	12-mo Change	Between-group difference
Control^b^	23.41 (6.34)	25.26 (5.73)	24.79 (6.15)	0.29 (−0.65 to 1.23)	NA	−0.39 (−1.17 to 0.39)	NA
Multiple imputation^c^	NA	NA	NA	1.42 (0.57 to 2.28)	NA	1.49 (0.75 to 2.22)	NA
Intervention^b^	22.87 (6.78)	26.20 (6.15)	26.68 (6.42)	2.30 (1.23 to 3.37)	2.01 (0.62 to 3.40)	2.28 (0.98 to 3.57)	2.67 (1.14 to 4.20)
Multiple imputation^c^	NA	NA	NA	2.57 (1.64 to 3.49)	1.14 (−0.10 to 2.38)	2.95 (1.94 to 3.95)	1.46 (0.21 to 2.72)

Abbreviation: NA, not applicable.

^a^ Multilevel mixed-effects linear regressions for repeated measures (baseline, 6-month, and 12-month study visits) and study group (intervention vs control) on mMDS changes at 6-month and 12-month follow-up and between-group differences are adjusted for age, sex, fire department, physical activity, race and ethnicity, and waist circumference. Fire stations (52 clusters) and participants (484 firefighters) were specified as the random effects parameters.

^b^ Total observations for baseline (n = 484), 6 months (n = 335), and 12 months (n = 260) vs imputed data sets for baseline, 6-month, and 12-month values (n = 484).

^c^ Multiple imputation results are pooled estimates from each of the 25 completed data sets comprised of observed and imputed values.

**Table 3.** Between-Group Differences for 6-Month and 12-Month Changes in Modified Mediterranean Diet Score (mMDS) Items by Study Group

mMDS Item	Observed mean points (95%CI)^a^		
	Control	Mediterranean nutrition intervention	Between-group differences^a^
**Fast-food or take-out food (0-4 points)**			
Baseline score, mean (SD)	2.22 (1.07)	2.26 (0.99)	NA
6-mo Change	−0.03 (−0.18 to 0.12)	0.10 (−0.08 to 0.27)	0.12 (−0.11 to 0.35)
12-mo Change	0.05 (−0.07 to 0.18)	0.28 (0.11 to 0.45)	0.23 (0.02 to 0.44)
**Fruits (0-4 points)**			
Baseline score, mean (SD)	1.68 (0.85)	1.58 (0.83)	NA
6-mo Change	0.01 (−0.09 to 0.11)	0.16 (0.01 to 0.32)	0.15 (−0.03 to 0.34)
12-mo Change	−0.04 (−0.15 to 0.06)	0.12 (−0.08 to 0.32)	0.17 (−0.05 to 0.38)
**Vegetables (not including potatoes) (0-4 points)**			
Baseline score, mean (SD)	2.13 (0.97)	1.96 (0.86)	NA
6-mo Change	−0.06 (−0.25 to 0.14)	0.18 (0.03 to 0.32)	0.23 (0 to 0.47)
12-mo Change	0.04 (−0.13 to 0.20)	0.19 (0.01 to 0.36)	0.15 (−0.09 to 0.39)
**Sweet desserts (eg, cake, cookies, pie, ice cream) (0-4 points)**			
Baseline score, mean (SD)	1.99 (1.52)	2.14 (1.48)	NA
6-mo Change	0.05 (−0.20 to 0.31)	0.15 (−0.08 to 0.38)	0.10 (−0.24 to 0.43)
12-mo Change	0.11 (−0.11 to 0.34)	0.35 (0.08 to 0.62)	0.24 (−0.11 to 0.59)
**Primary cooking oil/fat use at home (0-5 points)**			
Baseline score, mean (SD)	2.79 (1.96)	2.71 (2.05)	NA
6-mo Change	0.33 (0.05 to 0.61)	0.55 (0.20 to 0.91)	0.22 (−0.21 to 0.66)
12-mo Change	0.13 (−0.24 to 0.50)	0.32 (−0.19 to 0.83)	0.19 (−0.42 to 0.81)
**Primary cooking oil/fat use at work (0-5 points)**			
Baseline score, mean (SD)	2.65 (1.81)	2.52 (1.93)	NA
6-mo Change	0.42 (0.17 to 0.66)	0.55 (0.19 to 0.91)	0.14 (−0.29 to 0.56)
12-mo Change	0.02 (−0.26 to 0.30)	0.55 (0.15 to 0.96)	0.53 (0.04 to 1.02)
**Fried foods (eg, French fries, fried chicken, chicken nuggets) (0-4 points)**			
Baseline score, mean (SD)	1.92 (1.27)	2.07 (1.12)	NA
6-mo Change	0.10 (−0.04 to 0.24)	0.20 (0.01 to 0.38)	0.09 (−0.14 to 0.33)
12-mo Change	0.06 (−0.11 to 0.22)	0.23 (−0.02 to 0.47)	0.17 (−0.12 to 0.46)
**Breads/starches consumed at home (0-4 points)**			
Baseline score, mean (SD)	1.93 (1.74)	1.90 (1.80)	NA
6-mo Change	0.10 (−0.18 to 0.37)	0.36 (0.05 to 0.66)	0.26 (−0.14 to 0.65)
12-mo Change	0.07 (−0.22 to 0.36)	0.10 (−0.26 to 0.46)	0.03 (−0.44 to 0.49)
**Breads/starches consumed at work (0-4 points)**			
Baseline score, mean (SD)	1.59 (1.70)	1.42 (1.67)	NA
6-mo Change	0.15 (−0.16 to 0.47)	0.76 (0.46 to 1.07)	0.61 (0.20 to 1.03)
12-mo Change	0.23 (−0.18 to 0.64)	0.92 (0.40 to 1.43)	0.69 (0.03 to 1.34)
**Baked, broiled, grilled, or blackened ocean fish (eg, salmon, tuna, cod) (0-4 points)**			
Baseline score, mean (SD)	1.33 (0.91)	1.29 (0.97)	NA
6-mo Change	−0.01 (−0.17 to 0.15)	0.15 (0.02 to 0.28)	0.16 (−0.04 to 0.36)
12-mo Change	−0.06 (−0.19 to 0.08)	0.27 (0.13 to 0.42)	0.33 (0.14 to 0.53)
**Quantity of alcoholic beverages (0-4 points)**			
Baseline score, mean (SD)	2.08 (1.58)	2.14 (1.54)	NA
6-mo Change	0.09 (−0.04 to 0.21)	0.04 (−0.14 to 0.21)	−0.05 (−0.26 to 0.16)
12-mo Change	−0.03 (−0.21 to 0.15)	−0.31 (−0.54 to −0.08)	−0.28 (−0.57 to 0)
**Wine consumption (0-2 points)**			
Baseline score, mean (SD)	0.44 (0.83)	0.37 (0.78)	NA
6-mo Change	0.01 (−0.09 to 0.11)	−0.01 (−0.08 to 0.07)	−0.01 (−0.14 to 0.11)
12-mo Change	−0.01 (−0.15 to 0.14)	−0.02 (−0.10 to 0.06)	−0.01 (−0.17 to 0.15)
**Nonalcoholic beverages at home (0-4 points)**			
Baseline score, mean (SD)	2.86 (1.35)	2.72 (1.44)	NA
6-mo Change	−0.43 (−0.69 to −0.17)	−0.16 (−0.36 to 0.03)	0.27 (−0.06 to 0.60)
12-mo Change	−0.46 (−0.87 to −0.04)	0.05 (−0.37 to 0.47)	0.51 (−0.07 to 1.09)
**Nonalcoholic beverages at work (0-4 points)**			
Baseline score, mean (SD)	3.00 (1.23)	2.93 (1.33)	NA
6-mo Change	−0.34 (−0.66 to −0.01)	−0.18 (−0.47 to 0.10)	0.15 (−0.28 to 0.58)
12-mo Change	−0.56 (−0.83 to −0.29)	−0.22 (−0.69 to 0.24)	0.34 (−0.20 to 0.87)
**Legumes (eg, beans, chickpeas, lentils) (0-4 points)**			
Baseline score, mean (SD)	0.83 (1.12)	0.72 (0.99)	NA
6-mo Change	0.15 (−0.02 to 0.31)	0.10 (−0.01 to 0.20)	−0.05 (−0.25 to 0.15)
12-mo Change	−0.02 (−0.18 to 0.14)	0.14 (0.01 to 0.26)	0.15 (−0.05 to 0.36)
**Nuts (eg, walnuts, almonds, hazelnuts, pistachio, peanuts) (0-4 points)**			
Baseline score, mean (SD)	1.28 (1.36)	1.13 (1.26)	NA
6-mo Change	0.04 (−0.18 to 0.26)	0.26 (0.09 to 0.43)	0.23 (−0.05 to 0.50)
12-mo Change	−0.01 (−0.15 to 0.12)	0.27 (0.08 to 0.47)	0.29 (0.06 to 0.52)

Abbreviation: NA, not applicable.

^a^ Multilevel mixed-effects linear regressions for repeated measures (baseline, 6-month, and 12-month study visits) and study group (intervention vs control) on mMDS changes at 6-month and 12-month follow-up and between-group differences are adjusted for age, sex, fire department, physical activity, race and ethnicity, and waist circumference. Fire stations (52 clusters) and participants (484 firefighters) were specified as the random effects parameters.

### Secondary Outcomes

Among secondary end points, the Mediterranean nutrition intervention group experienced a significantly greater decline in body fat percentage by 0.79 absolute percentage points (95% CI, 0.18-1.40) and LDL cholesterol by −6.92 mg/dL (95% CI, −13.58 to −0.27) at 6 months compared with controls (Table 4). Other differences in cardiometabolic parameters were not statistically significant. Correction for multiple comparisons resulted in a loss of statistical significance (eTable 3 in Supplement 2).

**Table 4.** Between-Group Differences for 6-Month and 12-Month Changes in Cardiometabolic Parameters by Study Group

Cardiometabolic parameter	Mean (SD)			6-mo Follow-up, mean points (95% CI)^a^		12-mo Follow-up, mean points (95% CI)^a^	
	Baseline	6-mo	12-mo	6-mo Change	Between-group differences	12-mo Change	Between-group differences
BMI							
Control	30.14 (4.43)	30.06 (4.75)	30.20 (4.69)	0.60 (0.29 to 0.90)	−0.20 (−0.57 to 0.18)	−0.26 (−0.50 to −0.01)	−0.13 (−0.48 to 0.22)
Mediterranean nutrition intervention	29.92 (4.40)	29.93 (4.41)	29.57 (4.26)	−0.40 (0.18 to 0.61)		−0.38 (−0.64 to −0.13)	
Body fat percentage							
Control	27.61 (6.38)	27.87 (6.67)	28.27 (7.08)	0.87 (0.42 to 1.31)	−0.79 (−1.40 to −0.18)	−0.24 (−0.70 to 0.23)	−0.59 (−1.25 to 0.08)
Mediterranean nutrition intervention	28.67 (6.64)	28.15 (6.49)	27.42 (6.53)	0.08 (−0.37 to 0.53)		−0.82 (−1.29 to −0.36)	
Waist circumference							
Control	101.16 (12.48)	98.62 (12.39)	101.55 (12.56)	−2.42 (−3.51 to −1.33)	0.90 (−0.70 to 2.50)	1.24 (0.31 to 2.16)	−0.10 (−1.66 to 1.47)
Mediterranean nutrition intervention	98.27 (12.48)	97.52 (12.45)	98.38 (11.93)	−1.52 (−2.70 to −0.34)		1.14 (−0.16 to 2.43)	
Total cholesterol^b^							
Control	195.07 (38.25)	195.74 (36.86)	189.75 (36.26)	2.58 (−3.23 to 8.40)	−4.57 (−13.67 to 4.54)	−1.47 (−9.88 to 6.93)	3.16 (−7.58 to 13.90)
Mediterranean nutrition intervention	198.44 (36.58)	196.11 (39.81)	201.56 (35.87)	−1.98 (−9.04 to 5.08)		1.69 (−5.10 to 8.47)	
HDL cholesterol^b^							
Control	48.19 (11.51)	47.87 (12.77)	47.09 (11.33)	−1.83 (−3.57 to −0.08)	2.13 (−0.54 to 4.79)	−0.70 (−2.11 to 0.71)	1.26 (−0.81 to 3.34)
Mediterranean nutrition intervention	49.01 (11.17)	49.22 (12.48)	49.27 (13.45)	0.30 (−1.68 to 2.29)		0.56 (−0.99 to 2.11)	
LDL cholesterol^b^							
Control	122.05 (33.42)	122.63 (31.99)	117.99 (33.18)	3.05 (−1.17 to 7.27)	−6.92 (−13.58 to −0.27)	−0.28 (−6.87 to 6.32)	1.80 (−7.53 to 11.12)
Mediterranean nutrition intervention	124.36 (31.40)	120.51 (30.27)	127.74 (29.26)	−3.88 (−8.93 to 1.17)		1.52 (−5.11 to 8.15)	
Glucose^b^							
Control	98.97 (18.99)	99.53 (20.11)	100.82 (28.41)	−0.89 (−3.07 to 1.29)	1.54 (−3.64 to 6.72)	−0.17 (−3.04 to 2.69)	3.45 (−3.13 to 10.03)
Mediterranean nutrition intervention	99.97 (19.85)	101.19 (32.27)	98.76 (19.69)	0.65 (−4.49 to 5.79)		3.28 (−2.51 to 9.07)	
Triglycerides^b^							
Control	125.99 (73.63)	129.10 (105.92)	130.21 (98.21)	11.24 (−18.42 to 40.89)	−5.96 (−38.03 to 26.11)	3.65 (−21.05 to 28.35)	−6.66 (−38.37 to 25.04)
Mediterranean nutrition intervention	124.65 (78.57)	153.36 (329.90)	122.49 (82.95)	5.28 (−7.24 to 17.80)		−3.01 (−23.19 to 17.16)	

Abbreviations: BMI, body mass index; HDL, high-density lipoprotein; LDL, low-density lipoprotein.

SI conversion factor: To convert cholesterol to mmol/L, multiply by 0.0259; glucose to mmol/L, multiply by 0.0555; triglycerides to mmol/L, multiply by 0.0113.

^a^ Multilevel mixed-effects linear regressions for repeated measures (baseline, 6-month, and 12-month study visits) and study group (intervention vs control) on modified Mediterranean diet score changes at 6-month and 12-month follow-up and between-group differences are adjusted for age, sex, fire department, physical activity, and race and ethnicity but not waist circumference in the corresponding model. Fire stations (52 clusters) and participants (484 firefighters) were specified as random effects parameters.

^b^ Glucose and lipid profiles were determined using fasting blood samples collected during fire department medical examinations.

### Multiple Imputation

Results from the multiple imputation analyses were similar to the primary analysis. The mean change in mMDS for the Mediterranean nutrition intervention group was 1.14 points (95% CI, −0.10 to 2.38) greater than the control group comparing baseline with 6 months and 1.46 points (95% CI, 0.21-2.72) greater comparing baseline with 12 months (Table 2). Evaluating components of the Mediterranean diet, we found that multiple imputation showed greater improvements in Mediterranean nutrition intervention vs control in increased vegetable, fish, and legume consumption and improved nutritional quality of nonalcoholic beverages at home (eTable 2 in Supplement 2). Similar to the primary analysis, the imputation analysis showed a −1.03% (95% CI, −1.82 to −0.23) decrease in body fat percentage at 6 months in the Mediterranean nutrition intervention group compared with the control group (eTable 4 in Supplement 2), while LDL cholesterol was no longer statistically significant (−4.19%; 95% CI, −10.27 to 1.89).

Sensitivity findings based on influence analysis suggested that no single imputation estimate substantially influenced the pooled results (eFigure 1 in Supplement 2). Additionally, descriptive quality checks using histograms, kernel density plots, and summary statistics showed that the imputation models yielded reasonable imputed values (eFigures 2 and 3 and eTable 6 in Supplement 2).

## Discussion

In this 1-year cluster-randomized clinical trial, we found that a multicomponent behavioral/environmental Mediterranean nutrition intervention significantly improved Mediterranean diet adherence, increasing mean mMDS by 2.67 points (95% CI, 1.14-4.20), a more than a 10% increase from baseline, among career firefighters. Additionally, significant improvements were observed for several individual Mediterranean diet components. The results suggest that the workplace intervention strategy used in this trial may be an effective approach for improving nutrition among US career firefighters, who frequently experience atherosclerosis, left ventricular abnormalities, sudden cardiac death, and an excess of obesity-associated cancers.^4,16,39,40,41^

Few nutritional interventions within workplace settings have targeted firefighter populations.^42,43^ A recent randomized clinical trial among 137 San Diego firefighters with 24-hour shifts, the Healthy Heroes Study,^44^ demonstrated the feasibility of a Mediterranean diet with a self-selected 10-hour time-restricted eating window for 12 weeks and benefit on very LDL particle size (−1.10 nm; 95% CI, −2.16 to −0.03) compared with a standard of care arm. However, the sample size was relatively small, and both groups received the Mediterranean diet, since the intervention was testing time-restricted eating. Another recent cluster randomized clinical trial including 175 first responders from Massachusetts and Arizona demonstrated an improvement in CVD risk factors, primarily 1-year changes in α1 HDL cholesterol, body weight, waist circumference, triglycerides, and HDL to triglyceride ratio, through personalized diet and exercise plans given to each intervention participant.^14^ Currently, the 6-month FIREHOUSE randomized clinical trial in the Fire Department of New York is evaluating whether a restricted-calorie Mediterranean diet with behavioral/nutritional/exercise monitoring coupled with feedback and group counseling can alter metabolic biomarkers.^45^

Among different components of the Mediterranean diet, most suggested numeric improvements in the intervention vs control group, with statistically significant improvements in the quality of cooking oil and bread/starches consumed at work, increased fish and nut consumption, and fewer alcoholic beverages and fast food. Studying the individual Mediterranean diet components may help target simple dietary substitutions to improve diet quality among the US fire service.^39,46,47^

The implementation of the current trial across multiple firefighters’ worksite settings and routines yielded pragmatic and methodological advantages.^48,49^ One strength was engaging the firefighters in both their work and home environments, given that 80% of representative firefighters previously reported their spouse’s opinion/support was important for healthy eating, whereas almost one-third of weekly meals were consumed at the firehouse.^19,28^ Given the team setting has shown a greater influence on interventions focused on improving firefighter diet and eating together at the firehouse has been positively associated with benefits unique to the workplace,^43,50^ the evaluation specifically considered habits both in the firehouse and at home.

### Limitations

This study has limitations. As in many real-world behavioral interventions, a primary limitation was loss to follow-up. This was addressed by conducting multiple imputation analyses, which help correct the potential bias due to informative missing data and may offer more reliable and applicable results within clinical practice.^51^ The multilevel multiple imputation approach further ensured the compatibility of the analysis and imputation models, providing robust estimates that further supported our primary findings.^8^ Although dietary intake data were self-reported using 2 different instruments at baseline, the mMDS questionnaire was validated against a separate, detailed FFQ.^30,31^ Furthermore, provided that free food samples may facilitate accessibility and affordability, the provision of free samples may have increased adherence. Future studies may consider additional objective end points to increase statistical power to detect biometric improvements, such as continuous glucose monitors, wrist-worn actigraphy devices, and mobile apps that estimate a range of cardiometabolic and other health parameters.^52^ Future studies with longer follow-up and repeated measures of mMDS should also consider hard clinical outcomes to better understand the clinical relevance of our findings. Our mostly male, mostly White trial population is representative of Midwestern firefighters,^53^ although nearly 1 in 5 participants were Black or another race or ethnicity. Our results support the need for future studies to assess the generalizability of this intervention to other settings, such as more urban and more racially and ethnically diverse fire departments.

## Conclusions

This study tested the effectiveness of a multicomponent worksite intervention focused on adherence to the Mediterranean diet in modifying the dietary habits and risk factors of US career firefighters. At 1 year, firefighters in the Mediterranean nutrition intervention intervention group had significantly improved dietary habits compared with the control group. Given the high rates of diet-related mortality in this population, these novel results support the need for additional similar studies in other more diverse populations of firefighters to replicate and assess the generalizability of these findings.

## Notes

Supplement 1.
Trial Protocol

Supplement 2. **eTable 1.** Baseline Characteristics of Feeding America’s Bravest Participants by Follow-Up Completion **eTable 2.** Multiple Imputation Between-Group Differences for 6- and 12-Month Changes in mMDS Items **eTable 3.** Between-Group Differences for 6- and 12-Month Changes in Cardiometabolic Parameters and 95% CIs Corrected for Multiple Comparisons **eTable 4.** Multiple Imputation Between-Group Differences for 6- and 12-Month Changes in Cardiometabolic Parameters **eTable 5.** Description of the Modified Mediterranean Diet Scoring System **eFigure 1.** Influence Analysis Shows the Influence of Each Imputation Model Estimate on the Overall Effect Size Estimates for A) 6-Month and B) 12-Month Between-Group Differences in mMDS Changes **eFigure 2.** Quality Check for Multilevel Multiple Imputation Model #1; Histograms Show a Modified Mediterranean Diet Score (0-51 Points) for the Completed vs Observed Datasets and Mean (SD) by Study Group **eFigure 3.** Quality Check for Multilevel Multiple Imputation Model #1; Kernel Density Plots Show Observed, Completed, and Imputed Modified Mediterranean Diet Score Values for 6- and 12-Month Follow-Up **eTable 6.** Quality Check for Multilevel Multiple Imputation Model #1; Summary Statistics Compare Observations With Completed Dataset Values for the Modified Mediterranean Diet Score at 6- and 12-Month Follow-Up **eMethods.** Original Study Design and Intervention

Supplement 3.
Data Sharing Statement
