# Supplementary material for: Effect of a Nutrition Intervention on Mediterranean Diet Adherence Among Firefighters: A Cluster Randomized Clinical Trial
Source: JAMA Netw Open. 2023 Aug 17;6(8):e2329147. doi: 10.1001/jamanetworkopen.2023.29147 (PMC10436136; doi:10.1001/jamanetworkopen.2023.29147)
Supplement: Supplement 1. — Trial Protocol [file jamanetwopen-e2329147-s001.pdf]

Instructions: The purpose of this research protocol is to provide IRB members and reviewers with sufficient information to conduct a substantive review. If a separate sponsor's protocol exists, submit it in addition to this document.

Complete all of the sections below. For more detailed instructions, consult the Investigator's Manual or IRB website (links provided below).

GENERAL INFORMATION	
**Protocol #** (if assigned): IRB16-0170	
**Version Date:12/8/2017**	**Version Number: 6**
**Principal Investigator: Stefanos N Kales**	
**Faculty Advisor** (if PI is a student)**:**	
**Protocol Title: Feeding America's Bravest: Mediterranean Diet-Based Interventions to change Firefighters' Eating Habits and Improve Cardiovascular Risk Profiles**	

1. Specific Aims

1. Develop a multi-pronged, Mediterranean Diet Nutritional Intervention (MDNI) behavior change strategies including: diet/lifestyle education; discounted access to key Mediterranean diet foods; electronic education platforms and reminders. MDNI components will be refined via surveys, literature review and local/national firefighter input including labor/management and fire service focus groups.

Hypothesis (1). Cost-effective, fire-service MDNIs will be developed for the cluster-randomized, career firefighter diet intervention.

2. Cluster Randomized Diet Intervention-Phase I- the Indianapolis Fire Department (IFD) has over 1,000 members. All 44 IFD firehouses will be cluster randomized into two groups and then individual members will be consented regarding study participation. Group 1 will receive an active 12-month MDNI, while Group 2 will receive no intervention. Firefighters categorized as "Administrative Staff" by the IFD will also be approached for participation and if they consent, be assigned to Group 1 because they are not assigned to specific firehouses.

3. Cluster Randomized Diet Intervention -Phase II: Group 1 will cross-over to "self-sustained continuation" for 12 months to examine persistence of behavior change during this less intense, self-directed maintenance diet intervention. Group 2 will cross-over to receive the MDNI for 6 months (assessing a shorter MDNI), followed by a final 6 months of self-sustained continuation. An additional neighboring fire department, Fisher's (IN), will have its 6 firehouses randomized to receive the MDNI (Group 3) or no intervention (Group 4) for six months, and then individual members will be consented regarding study participation. After 6 months, the 3 stations in the MDNI (Group 3) will cross-over to "self-sustained continuation" for the last 6 months to examine persistence of behavior change during this less intense, self-directed maintenance diet intervention. Group 4 will cross-over to receive the MDNI for 6 months (assessing a shorter MDNI).

4. A similar 12-month Cluster Randomized Diet Intervention in the six fire stations of the Fishers (IN) Fire Department with a cross-over by the control group to intervention after 6 months will also be done.

Our existing modified Mediterranean diet score (mMDS) will be further integrated with other validated scores and nutrition questionnaires. Questionnaires, mMDS and clinical data will be collected longitudinally throughout both phases of the Nutritional Intervention. The complete questionnaire has been entered into Qualtrics and is attached for the IRB's review.

Hypotheses (2-3). Both MDNI lengths will improve mMDS, reduce weight, and improve Cardiovascular Disease (CVD) risk profiles in career firefighters. The 12 month MDNI will produce greater persistence of adherence than a 6 month MDNI. Cost-effective methods that combine messaging and discounts for improving firefighters' diets will be developed and validated, and these low cost methods will be adaptable for widespread translation and implementation throughout the career fire service.

5. a. Conduct a nested evaluation of adherence biomarkers (urine tyrosol and hydroxityrosol and plasma fatty acids) among randomly selected participants in the two Indianapolis MDNI arms (MD intervention vs. control, up to 100 in each).

5.b. Blood samples will also be stored for future analyses.
5.c. Stool samples will also be stored for microbiome analyses.

2. Background

2.1 Provide the scientific background and rationale for the study

Cardiovascular disease (CVD) is the leading cause of on-duty death (about 45% of on-duty fatalities) and lifetime mortality (at least 30% of all deaths), as well as morbidity among US firefighters. Thus, interventions which prevent CVD are clearly the top priority for US fire service research. The effectiveness of the Mediterranean diet in preventing CVD is roughly equivalent to statin medications (e.g. Lipitor, Crestor), but without side effects; while providing added benefits on weight control, diabetes prevention and decreased cancer risk, however, there is little available experience with worksite implementation of Mediterranean diet.

Obesity and cardio-metabolic risk clustering are well-established CVD risk factors. Current evidence demonstrates a growing obesity problem in the fire service, with obesity to have documented adverse effects on: fitness, metabolic syndrome, CVD events, including SCD; injury risks/workers' compensation costs; job-related disability and CVD retirements.

Existing Dietary Challenges and Opportunities: Various factors contribute to the high prevalence of obesity and CVD risk clustering in the fire service, including: shift work-induce sleep disturbance/sleep deprivation; unreliable meal times which increase the risk of less optimal dietary choices and greater consumption of fast-/takeout-foods; and traditions around over-eating and less healthy food choices common in fire service culture. According to our recent study, the two dietary factors that differed the most between obese and non-obese firefighters were obese firefighters' higher consumption of sugary drinks and fast-food.

Mediterranean Diet on CVD and Cancer risk reduction: Mediterranean-style diets are similar eating habits traditionally followed in countries bordering the Mediterranean Sea. The general pattern is characterized by high consumption of olive oil, fruits, vegetables, unrefined (whole grain) breads and cereals, legumes and nuts; moderate consumption of fish and poultry; a relatively low intake of dairy products; and low-sparing consumption of red meat, processed meats and sweets.

Numerous studies have demonstrated the effectiveness of Mediterranean diets in reducing all-cause mortality, CVD morbidity and mortality and cancer mortality. The mechanisms are likely mediated through beneficial effects on CVD risk factors such as obesity, hypertension, diabetes mellitus and metabolic syndrome. Given these clear benefits, the latest US government nutritional guidelines recognize and recommend the Mediterranean Diet as one of three healthy options for Americans.

Mediterranean Diet and Workplace Behavioral Change: As we recently summarized in a published review of Mediterranean diet and the workplace, experience with worksite MDNIs is limited, but the evidence is quite positive. Shai et al. conducted the only randomized controlled trial at an Israeli nuclear facility. Over 300 obese participants were randomly assigned to: a low-fat, restricted-calorie diet; a Mediterranean, restricted-calorie diet;

or a low- carbohydrate diet without calorie restriction. After 2 years, the mean weight loss was 2.9 kg in the low-fat group, 4.4 kg in the Mediterranean group, and 4.7 kg in the low carbohydrate group. The participants were then followed for an additional 4 years without active intervention. After 6-years, the total weight loss was greatest and most significant for the Mediterranean group (3.1 kg), whereas the other two groups gained back most of their weight.

Preliminary cross-sectional data among firefighters showed that greater adherence to a Mediterranean dietary pattern as measured by the modified Mediterranean diet score (mMDS) was significantly associated with improvements in body fat, metabolic syndrome, LDL- and HDL-cholesterol, weight, and aerobic fitness. Dietary modification is more likely to be effective when the strategy is appealing to the target population and addresses perceived knowledge gaps. A national survey that our group conducted demonstrated that a majority of career firefighters (71%) do not currently follow any particular dietary plan, feeling that they receive insufficient information from the fire service on nutrition, while they are interested in learning more about nutrition and healthy eating and over 60% wanted to learn more about the Mediterranean diet. The survey rankings demonstrate that while most firefighters reject a strictly plant-based diet, they are very receptive to a Mediterranean diet and rank it the highest.

Biomarkers and diet evaluation of food or nutrient intake are directly measured and can objectively estimate the consumption of specific dietary items without self-reporting biases and inaccuracies. Thus, by randomly assessing the adherence biomarkers within both the MD and control arms subsets of firefighters participating in the cluster-randomized controlled trial (Feeding America's Bravest), we can also better determine the validity of our dietary questionnaires with respect to Mediterranean diet-desirable fats. Blood samples can be used for further metabolomics analyses that will help us develop short-term indicators for assessing the effect of nutritional changes.

In summary, the negative effects of obesity and CVD on the fire service are recognized, but few effective preventive programs exist, and behavior change strategies must be improved. Evidence suggests that a Mediterranean diet nutrition intervention would be effective and well-received by career firefighters.

2.2 Describe the significance of the research, and how it will add to existing knowledge

Beyond the previous background and justifying the need of the proposed project, the research will have additional significance and impact. Our research would expand the literature on the use of Mediterranean diet interventions in the workplace and among non-Mediterranean populations. A successful intervention would suggest that our approach could be disseminated and implemented nationally in the fire service, as well as potentially among other public safety professions such as law enforcement.

3. Study Setting

3.1. Identify the sites or locations where the research will be conducted.

Indianapolis Fire Department and Fishers Fire Department.

3.2. Describe the Principal Investigator's experience conducting research at study site(s) and familiarity with local culture

Dr. Kales is a Professor of Medicine at Harvard Medical School, and Professor/Director of the Occupational Medicine Residency at the Harvard TH Chan School of Public Health. He is Division Chief of Occupational Medicine at the Cambridge Health Alliance, a Harvard- affiliate and is Board Certified in Occupational Medicine. Dr. Kales' clinic provides clinical services to fire and EMS personnel, and he has worked with the fire service (both management and labor, as well as national foundations) regionally and nationally for over 20 years. His research group has provided landmark contributions related to CVD among firefighters. Therefore, Dr. Kales is extremely familiar with fire service culture and well-respected within it.

Dr. Kales has ample experience and success in leading and managing grant-funded projects similar to the proposed research; and as the PI will direct all aspects of the current proposal. He has received Massachusetts, Federal (Career Award, NIOSH RO1, three prior FEMA R&D grants) and Canadian funding. Finally, Dr. Kales has worked closely with the Indianapolis Fire Department (IFD) since 2012 on another FEMA-funded study which is now finishing. He has visited the IFD on several occasions and has an excellent working relationship with the IFD Chief, union local and fire department medical director (Chief Medical Officer of Public Safety Medical in Indianapolis). The latter is a co-investigator on this proposal.

3.3. Is the research conducted outside the United States?

☒No ☐Yes: **If yes; describe site-specific regulations or customs affecting the research, local scientific and ethical review structure**

3.4 Are there any permissions that have been or will be obtained from cooperating institutions, community leaders, or individuals, including approval of an IRB or research ethics committee?

☐No ☒Yes: **If yes; provide a list of the permissions (also include copies with the application, if available)**

- Chief of Indianapolis Fire Department (IFD) and Indianapolis Local 416 (see attached letters of support). These letters offer the support of the IFD through its Chief and the union through its President (to which the firefighters belong) to participate in the proposed research at the department level. We also provide a similar letter of support from the Fishers (IN) Fire Department. Letter of support and commitment with the analyses of the nested biomarkers study are provided as well (from CSTI, University of Athens and Ohio University award letter to conduct the study.

University of Athens will received the specimens de-identified for urine biomarkers analysis.

Ohio University has already relied on this approved IRB protocol and the documentation is already uploaded.

4. Study Design

4.1. Describe the study design type

Prospective, cluster-randomized Mediterranean Diet Nutritional Intervention versus no nutritional intervention for 12 months; followed by cross-over of interventions for an additional 6-12 months. The additional participants from Fishers (IN) Fire Department will be studied for 12 months. The nested study for the evaluation of adherence biomarkers and the stool sample collection will last 6 months.

4.2. Indicate the study's duration - and the estimated date of study completion

2 years. December 2018

4.3. Indicate the total number of participants (if applicable, distinguish between the number of participants who are expected to be screened and enrolled, and the number of enrolled participants needed)

1,000 career firefighters (500 assigned to group 1 and 500 assigned to group 2). We will approach as many eligible members as possible to consent at least 500 and up to 1,000 participants maximum. For the biomarkers nested study, a total of up to 100 firefighters, selected at random evenly from the existing study arms in Indianapolis

4.4. List inclusion criteria

IFD and Fishers members eligible for study consent and participation will include those: a)

permanently assigned to one of the 44 IFD stations or one of the 6 Fishers stations; b) with a fire department-provided medical exam in the last two years; c) at least 18 years of age; and d) full duty, modified or restricted duty status at the time of consent; OR e) Firefighters categorized as "Administrative Staff" by the IFD or Fishers will also be approached for participation and if they consent, be assigned to Group 1 because they are not assigned to specific fire houses.

4.5. List exclusion criteria

IFD or Fishers members not eligible for study consent and participation will include those: a) without a recorded fire department exam in the last two years; b) less than 18 years of age.

4.6. Describe study procedures

Human subjects-trained staff from Public Safety Medical (PSM) working with the IFD/ Fishers Chiefs and firefighters' local union presidents will announce the inauguration of the study to IFD/ Fishers members and their opportunity to participate. Next, PSM staff will approach eligible IFD/ Fishers members for informed consent (see section 6.1).

Baseline Assessment:

- Using established coded data transfer methods between Public Safety Medical and the Harvard TH Chan School of Public Health. Public Safety Medical will transfer the results of the last existing fire department medical examination to the Harvard Chan research team for consented participants only.
- Medical exam data that will be provided to the study team for consented IFD/ Fishers subjects include: at rest and maximal heart rate, systolic and diastolic blood pressure, heart rate recovery, electrocardiographic findings at rest, hip and waist circumference, body composition, data regarding the grip strength, leg-press and push-ups performed, and routine lab values (e.g. lipid panels, hs-CRP and glucose).
- The medical exams will be supplemented by a comprehensive nutritional-lifestyle questionnaire (see attached). The survey instrument combines our existing mMDS with other validated scores and nutrition questionnaires. It also includes items on physical activity and other health behaviors to adjust for appropriate covariates. These questionnaires will be self-administered online via Qualtrics on a password-protected site. The comprehensive nutritional-lifestyle questionnaire should take between 20-45 minutes to complete.

In addition, the baseline assessment will include anthropometric measures (height, weight and body fat estimates (Tanita) and resting blood pressure will be taken in private areas of the fire houses by trained Public Safety Medial clinical staff, during work hours. Height will be measured in the standing position with a standard clinic stadiometer. Waist circumference will be assessed by using a tape measure snugly around each participant's waist at the level of the iliac crests and measuring the circumference after expiration. Body weight will be measured with bare feet and in light clothes on a calibrated scale. Body fat will be estimated using a Bioelectrical Impedance Analyzer (Tanita). Blood pressures will be measured using an appropriately sized cuff with the subject in the seated position in a resting state.

The participants in the biomarkers nested study will be already participating in the Feeding America's Bravest. The baseline collection time for the biomarkers coincide with the 1-year follow up. The second visit will be after 6 months of follow up or the original 18 months of follow up (see timeline in figure 1 and the second table below. Thus, the participants will be filling out the lifestyle/nutrition questionnaires/ anthropometrics and medical exam as explained previously in the protocol in each time point. The additional tests they will take are described below:

y,- Two EDTA blood collection tubes will be collected (<15ml of blood) in the fasting state (meaning no food or liquids except water for at least 12 hours before the appointment) at time point 12 and at 18 months of follow-up. Plasma and serum will be aliquoted, frozen at –80°C, stored and run in batches. Forty fatty acids will be analyzed including oleic, alphaE linolenic acid (w3), and alphaElinoleic (w6), which are reliable indicators of MUFA, tree nut and other healthy fat consumption. Analyses at time

point 1 and after 6 months will be assessed by gas chromatography at the Harvard T.H. Chan School of Public Health, which provides high quality laboratory measurements to studies around the globe. The rest of the plasma and blood samples collection will be stored for future potential studies at Ohio University in the Edison Biotechnology Institute.

-Urine samples will be collected at the same time as blood samples. First spot morning urine will be obtained at home, after an overnight fast, following specific instructions for its collection (same as those given for routine laboratory analyses). Hydroxytyrosol and tyrosol will be measured by gas chromatography–mass spectrometry by Professors Magiatis' experienced team at the University of Athens.

- Both blood and urine samples will be deidentified and coded, initially processed at a private company, Clinical and Translational Support (CTSL), Indianapolis. Blood samples will be kept cool and will be processed to separate plasma immediately after the withdrawal in a refrigerated centrifuge and then, working on ice, immediately aliquotting into cryovials and placing into -80°C storage. Shipping to the Furtado and Magiatis labs will be done on dry ice via FedEx overnight service in a cardboard and Styrofoam cold shipping container. The remaining aliquots will be sent and storage at Ohio University.

- Stool samples: Stools samples will be collected with OMNIgene®•GUT kit that is an all-in-one system for easy self-collection and stabilization of microbial DNA from feces for gut microbiome profiling. The participants will self-collect fecal samples according to the standard instructions provided with the kit. The kit includes the a two-way mailing box with pealable label. The stool is considered exempt human specimens, and can be sent via the normal postal system using their mailing package. Once the samples arrive at Ohio University they will be processed following the instructions: http://www.dnagenotek.com/ROW/pdf/PD-PR-00695.pdf

The samples will be collected in Indianapolis at Public Safety Medical by trained and qualify staff. The first step of sample preparation will be conducted in the Clinical and Translational Support CTSL Laboratory.

Follow-up Assessments:

Every consented participant (regardless of intervention assignment) will be requested to complete the following every 6 months over 4 additional visits after the baseline assessment over the two years of the study:

The comprehensive nutritional-lifestyle questionnaire will be repeated (detailed above), as will the anthropometric measures (height, weight and body fat and resting blood pressure) will be collected in the same manner as described above for the baseline assessment.

Every 12 months, the Fire department provided, annual occupational medicine examinations (part of the firefighters' usual care unrelated to the present study) will be provided by Public Safety Medical as per existing IFD/ Fishers policies. For consented participants ONLY, the medical exam results will be transferred to the Harvard Chan research team using the same established coded data transfer methods as described above for the baseline assessment of the same data points.

Consented participants will be assigned to Group 1 (Phase 1 nutritional intervention) or Group 2 (No intervention) according to their fire station's cluster randomization.

For the nested biomarkers study the recruitment of participants will start in phase II following the below timeline:

Figure1. Current parent study timeline and biomarkers proposal timeline (in orange, the proposed study timeline)

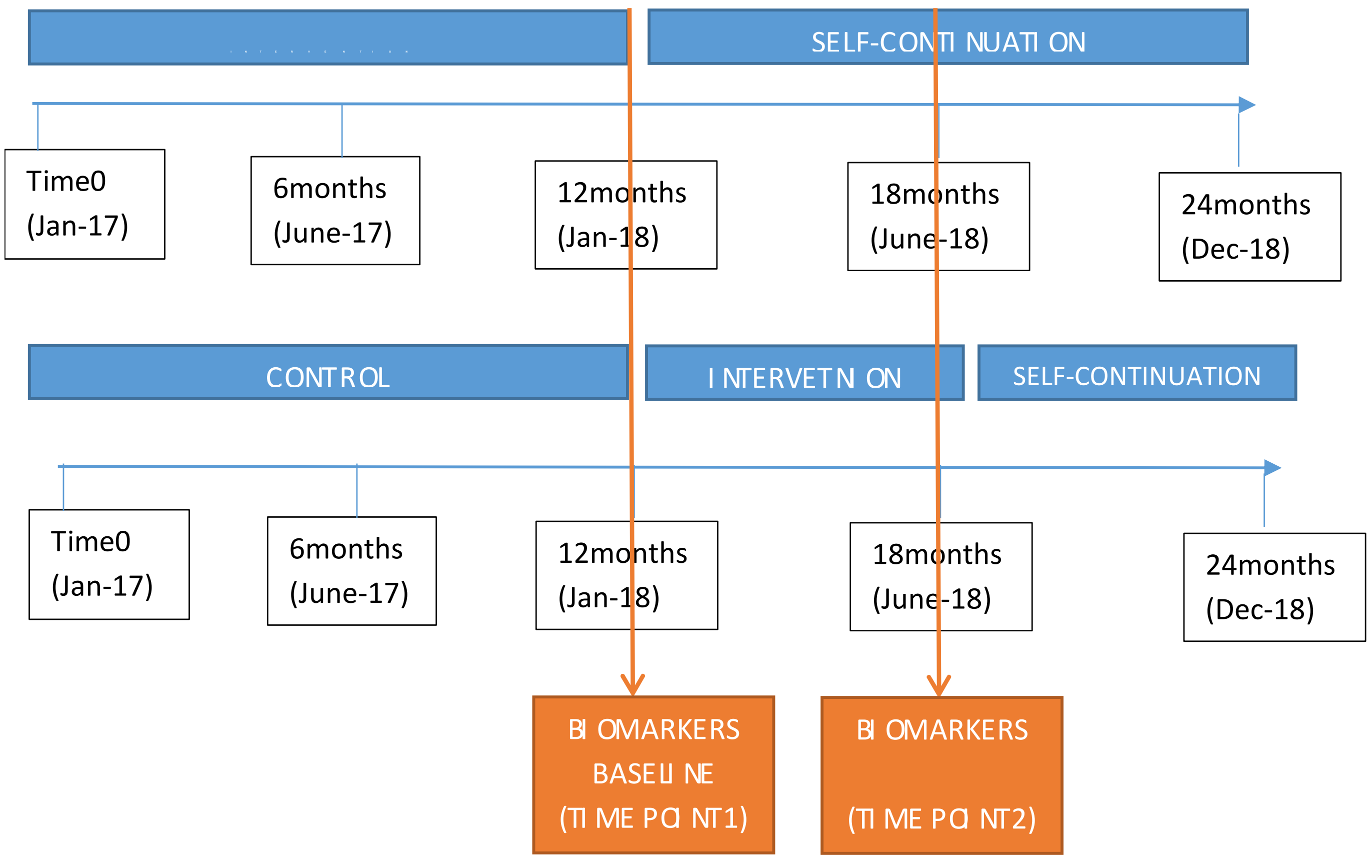

PHASE I (first 12 months of the study) Group 2: No intervention.

Mediterranean Diet Nutritional Intervention for 12 Months for Group 1. The extent to which consented participants personally engage in the MDNIs is voluntary, and the additional time commitment required for the study is nominal. The Mediterranean Diet intervention will include educational materials; opportunities for discounted access to healthy foods consistent with the Mediterranean diet (e.g. extra-virgin olive oil, nuts, fruits, vegetables, legumes, whole-unrefined grains, and lean proteins) for both participating firefighters and their families; peer education/support, on-line learning, and email and text message encouragement and reminders. Each of these pieces of the intervention is explained in further detail below.

Educational materials (online learning) will be provided via the study website; group educational sessions and written materials. Examples of our educational information include: a brochure with Mediterranean Diet recommendations, shopping list recommendations and sample recipes, tips to practice the Mediterranean Diet at both home and work and a firefighter-specific Mediterranean Diet Pyramid have been developed for this purpose. These materials may be supplemented as the study progresses to include additional modified Mediterranean Diet recipes based on the firefighters' and their families' preferences and requests (e.g. supply a modified recipe for healthy Mediterranean lasagna or chili based on a fire station's request); short videos; and links to other healthy eating materials. The videos may include: cooking demonstrations; talks with firefighter "champions" (brief interviews and commentaries from firefighters who are successfully practicing the Mediterranean diet) and similar content.

The initial and periodic group educational sessions with larger groups (up to 100 firefighters) will be led by the Harvard investigators and the local medical team (Public Safety Medical) and held in fire department classrooms. These will be followed-up by more frequent, smaller group (5-20 firefighters) diet and fitness sessions in fire house classrooms and kitchens by our Indiana-based dietician and Health/Fitness Specialist, Heather Fink. Ms. Fink is contracted with PSM and has a long experience working with firefighters. The sessions may include: didactic presentations, interactive discussions and cooking demonstrations that follow the general recommendations (see below).

Specific topics that we plan to address in the group educational sessions include:

1. Basic Mediterranean Diet principles;
2. The benefits of Olive oil and nuts and how to introduce them into the diet;
3. Overcoming Challenges while following a Mediterranean Diet;
4. How to plan a weekly menu and shopping list;
5. How to choose wisely when eating out (restaurants/parties);
6. Making smart choices regarding "Fast food".

We may also provide in-person chef-led, Mediterranean cooking demonstrations at fire stations in the intervention group. Some of the general educational sessions and cooking demonstrations may be videotaped or otherwise adapted for internet accessibility on the study website.

All of the educational sessions and materials will be centered on the principles and general recommendations outlined in the table below.

MEDITERRANEAN DIET: GENERAL RECOMMENDATIONS

FOOD	GOAL
**RECOMMENDATION**	
1. Olive oil (extra virgin if possible)	Main culinary fat or ≥4 tbsp/day
2. Tree nuts and peanuts (peanut butter without added sugars or hydrogenated fat)	≥3 servings/wk
3. Fresh fruit	≥3 servings/day
4. Vegetables	≥2 servings/day
5. Fish (including fatty fish) and seafood	≥3 servings/wk
6. Legumes (chickpeas, beans, lentils)	≥3 servings/wk
7. Sofrito[a]	≥2 servings/wk
8. Fresh Herbs, Allium (onion, garlic, etc)	≥2 servings/day
9. Yogurt	≤2 servings/day

10. White meat	Instead of red meat (2-3 servings/wk)
11. Whole grains/cereals[b]	Instead of refined grains
12. Wine with meals (optional, only for habitual drinkers)[c]	≥7 glasses/wk
**DISCOURAGE**	
13. Soda drinks (encourage water as a beverage)	<1 drink/day
14. Spread fats	<1 serving/day
15. Red and processed meats	<1 serving/day (< 2 servings/wk)
16. Commercial bakery foods sweets, and pastries[d]	<3 servings/wk
17. Fast food	≤1/wk

[a] Sofrito is a tomato-sauce made with onion and garlic, slowly simmered with olive oil
[b] Women 75 g/day, men 90 g/day or ≥5 servings/day. Whole grains brown rice, whole grain bread and pasta. [c] Optional and only for habitual drinkers without alcohol-related health problems
[d] Homemade sweets are preferable to store-bought or commercially-produced sweets

Peer-education and support: In addition, we will identify volunteers among consented participants at each fire house assigned to the nutrition intervention to become station "champions". These peer-nutrition leaders can help to motivate their colleagues and also report back to the investigators regarding which strategies are most successful as well as on obstacles encountered.

Discounted food access: Among the most important perceived barriers to healthy eating are the time and cost of shopping. Therefore, we have partnered with Kroger supermarkets, a large national chain with numerous stores in the Indianapolis area, to provide discounted access to key Mediterranean foods (eg extra-virgin olive oil, nuts, fruits, vegetables, legumes, whole-unrefined grains, and lean proteins) for both participating firefighters and their families. The discount program within the study will utilize Kroger's existing shopper loyalty program which constantly features discounted items and promotions for registered customers. All participating firefighters will be encouraged to register with the program if not already members. On a regular periodic basis (e.g. 1-2 times per month), Kroger staff will identify and "package" existing store discounts, price promotions and coupons that are consistent with the Mediterranean Diet intervention into "mailers" which advertise and list the items and promotions. Copies of the mailers will be sent to the firehouses assigned to the active intervention. In this way, both firefighters and their families may be aware of the discounts and have the option to use them.

Kroger staff will use the following guidelines to select food items related to those highlighted in green here for inclusion in the mailers.

TABLE 1. General Dietary Recommendations Based on Mediterranean Diet Principles

Daily Intake		Weekly Intake		Sparing Intake	
**Food Item**	**Goal**	**Food Item**	**Goal**	**Food Item**	**Goal**
Olive oil	≥4 tbsp/day	Nuts	3–7 serv/week	Red and processed meat	≤2 serv/week
Yogurt	≤2 serv./day	Fish/seafood	≥3 serv/week	Commercial sweets and refined carbohydrates[§,||]	<3 serv/ week
Fresh fruits	≥3 serv/day	Eggs	2–3 serv/week	Soda and sugary drinks[§,||]	<1/day
Vegetables	≥3 serv/day	Poultry	2–3 serv/week		
Whole and refined grains[*]	Women 75 g/day, men 90 g/day	Legumes	≥3 serv/week		
Fresh Herbs, Allium (onion, garlic, etc)	≥twice/day	"Sofrito"[‡]	≥2 serv/week		
Wine with meals[†]	Men 1–2 glasses/day Women 1 glass/day				

[*]Whole grains brown rice, popcorn, and any grain food with a carbohydrate to fiber ratio no more than 10:1.
[†]Optional and only for habitual drinkers without alcohol-related health problems.
[‡]Sofrito is a sauce made with tomato and onion and/or garlic, slowly simmered with olive oil.
[§]Homemade sweets are preferable to store-bought or commercially-produced sweets.
[||]Discouraged.

JOEM • Volume 58, Number 3, March 2016

Participants may also have access to additional discounted food options during the course of the study as follows:

1. Discount coupons obtained directly fromproducers which can be redeemed at Kroger and other local stores. For example, Barilla pasta has pledged its support to provide coupons for "Barilla Plus", a line of pastas which combine ALA-3- enriched whole grain and legume flours to produce a high fiber, higher protein product yielding delicious pasta that is more nutritious and promotes better glucose metabolism than typical pasta from refined flours.

2. "Starter" supplies of extra-virgin olive oil, avocados and nuts and for fire house kitchens at no cost, along with coupons and options for obtaining additional product at discounted prices directly through food companies partnering with the study.

3. Encourage and facilitate access to farmer's markets and produce from local community gardens within the City of Indianapolis and surrounding area.

According to each participant's preference, they may sign up for either <u>email or text message encouragement and reminders during the intervention</u>. For those opting in to this option, they will receive brief text or email messages 2-4 times per week reinforcing the healthy dietary and lifestyle principles as discussed above.

PHASE II

Mediterranean Diet Nutritional Intervention for 6 months for Groups 2 and 3 (and 4 in the final 6 months).

This intervention will be exactly as described above for Phase I, but will last 6 months. Afterwards, over the final 6 months of the study, the participants from Group 2 in Phase 1 will be encouraged to maintain eating Mediterranean style on their own using the support of the discount program, the study website and the support of fellow firefighters and family.

Group 2: No intervention. Over the next 12 months (year two). Phase 1 Group 1 participants will be encouraged to maintain eating Mediterranean style on their own using the support of the discount program, the study website and the support of fellow firefighters and family.

The table below summarizes data collection from all consented firefighter participants across the timeline of the 2-year study. Fishers Department FF will be joined to the study in the 12 months of follow up of the original Feeding America's Bravest

Questionnaires/Measurements	Baseline	6months	12months	18months	24 months
Nutrition/Lifestyle questionnaires	X	X	X	X	X
Blood Pressure	X	X	X	X	X
Height/Weight	X	X	X	X	X
Waist circumference	X	X	X	X	X
Body composition (Tanita)	X	X	X	X	X
Medical exam results transfer	X		X		X

: .

The table below summarizes data collection from a random subset of consented firefighter participating in the biomarker study

Biomarker proposal	Time point1 (Dec2017/Jan 2018)	Time point2 June2018)
Blood sample collection (fatty acids measurements)	X	X
Urine sample collection (hydroxityrosol and tyrosol)	X	X
Stool samples	X	X

4.7. Does the study involve the collection of data/specimens (including the use of existing data/specimens)?

☐ No ☒Yes: If yes; indicate how, when, where and from whom specimens or data will be obtained

Fire department provided annual occupational medicine examinations (part of the firefighters' usual care) will be provided by Public Safety Medical as per existing IFD policies and without regard to study participation. For consented participants **ONLY,** medical exam and biomarkers results will be transferred to the Harvard Chan research team as described above, as coded data in password-protected files. Medical exam data that will be provided to the study team for consented IFD subjects include: at rest and maximal heart rate, systolic and diastolic blood pressure, heart rate recovery, electrocardiographic findings at rest, hip and waist circumference, body composition, data regarding the grip strength, leg-press and push-ups, and routine lab values (e.g. lipid panels, hs-CRP and glucose), biomarkers from blood and urine and microbiome composition from the stools..

4.8. Is there a data and safety monitoring plan (required for greater than minimal risk studies)?

☐No ☒Yes: If yes; describe the plan

The proposed research presents minimal risk because the Mediterranean diet is proven to be effective and safe. Nonetheless, because our aim is to register our nutritional intervention in order to publish in high-impact journals, a data advisory monitoring board (DAMB) has been selected based on the members' nutrition, clinical, methodologic, statistical, or fire service expertise. The DAMB members include: Dr. Demosthenes Panagiotakos (Professor in Biostatistics, Research Methods and Epidemiology of Nutrition, at the Department of Nutrition Science - Dietetics, Harokopio University in Athens) who has been a leader in statistics and cardiovascular and nutritional epidemiology; Dr.
Walker S. Carlos Poston (Senior Principal Investigator, Center for Fire, Rescue & EMS Health Research, Institute for Biobehavioral Health Research, National Development & Research Institutes, Inc.) with extensive experience in statistics, epidemiology and the United States fire service; and Dr. Stefania Papatheodorou (Lecturer in Public Health, Cyprus International Institute for Environmental and Public Health) who is an accomplished epidemiologist and statistician.

The DAMB will monitor interim data (described in Section 4.7) in coordination with our team's statistician (Prof. Costas Christophi and the study PI, Dr. Kales).

4.9. Are there any anticipated circumstances under which participants will be withdrawn from the research without their consent?

☒No ☐Yes: If yes; describe the circumstances as well any associated procedures to ensure orderly termination

5. Data/Statistical Analyses Plan

5.1. Briefly describe the plan for data analysis (including the statistical method if applicable)

Data recording, storage, management, cleaning and basic analyses will be performed using SPSS. The data will be imported into Stata and SAS for more advanced statistical analyses such as multivariable regression

and longitudinal data analysis, as needed. Differences in the mean values of a quantitative variable between two groups will be assessed using the independent t-test whereas differences in mean values among three or more groups will be examined using the analysis of variance (ANOVA) technique (or non-parametric Wilcoxon and Kruskal-Wallis tests, respectively, as appropriate). Differences in qualitative characteristics will be compared using the chi-square test, or Fisher's exact test, or McNemar's test (for paired comparisons), as appropriate. Statistical significance for all analyses will be $p < 0.05$, and all tests will be two-tailed.

Phase 1: During the first 12 months of the nutritional intervention, **groups 1 (Mediterranean Diet intervention) and 2 (no intervention) will be compared directly on an intention to treat basis according to the randomization of each participant's fire house.** As several outcomes will be assessed on each participant throughout the study (3 annual medical exams and 5 semi-annual assessments (where weight, mMDS, etc. will be evaluated)), repeated measures techniques, such as mixed modeling and generalized estimating equation models, will be utilized to estimate changes over time on key outcomes. We will take into consideration the specific variance-covariance structure of correlated measurements. After adjusting for baseline and time-dependent covariates, changes in the outcomes of interest will be assessed and compared among groups. We expect our greatest power to be for within-group changes (paired comparisons).

5.2. Is there a sample size/power calculation?

☐**No** ☒**Yes: If yes; describe the calculation and the scientific rationale, and, if applicable, by site and key characteristics such as participant demographics**

Sensitivity analyses have been performed and demonstrate that even in the worst case scenario where only 500 firefighters would be enrolled, the study is well-powered for intention to treat comparisons of Mediterranean Diet Nutritional Intervention (MDNI) vs no intervention. Several conservative examples are given below for very small changes. However, consistent with previous studies, we expect the MDNI to actually produce larger changes that would be more easily measured. The first example assumes the MDNI can achieve a small increase in the mMDS of 6 points (about 1.0 times the population's baseline SD based on our cross-sectional study of mMDS)

For the biomarkers study spearman regression coefficients will be calculated to assess the association between *1*) self-reported olive oil consumption, walnuts, tree nuts, other food groups of the Mediterranean Diet and the overall score on the modified Mediterranean Diet scale and the biomarkers. Linear regression models, adjusted for potential confounders will be fitted to study the association of previously mentioned foods and biomarkers. Changes in biomarkers and comparison between groups will be assessed using mixed modeling and generalized estimating equation models or other appropriate statistical tests depending upon the normality

Population			Mmds					
Total	MDNI	Usual Care	SD	Treated	Untreated	Difference	Alpha error	Power
500	250	250	5.6	27.3	21.3	+6.0	0.05	>99%
750	325	325	5.6	27.3	21.3	+6.0	0.05	>99%
1000	500	500	5.6	27.3	21.3	+6.0	0.05	>99%

The second example is for a small body fat change (1.7%) using typical values from our cross-sectional data

Population			Body Fat					
Total	MDNI	Usual Care	SD	Treated	Untreated	Difference	Alpha error	Power

500	250	250	6.6	22.7	24.4	-1.7	0.05	82%
750	325	325	6.6	22.7	24.4	-1.7	0.05	94%
1000	500	500	6.6	22.7	24.4	-1.7	0.05	98%

The third example is for the prevalence of metabolic syndrome. We have found a metabolic syndrome prevalence of about 28% among career firefighters.[41] Based on previous studies, we conservatively expect the MDNI to reverse at least 35% of cases.

Population			Metabolic Syndrome Prevalence					
Total	MDNI	Usual Care	% Δ	Treated	Untreated	Difference	Alpha error	Power
500	250	250	-35	18%	28%	-10.0%	0.05	76%
750	325	325	-35	18%	28%	-10.0%	0.05	90%
1000	500	500	-35	18%	28%	-10.0%	0.05	96%

6. Recruitment Methods

6.1. Does the study involve the recruitment of participants?

☐ **No: If no, skip to <u>7.1</u>**

☒ **Yes: If yes; indicate how, when, where, and by whom participants will be recruited**

6. **How:** Up to 1000 IFD members with permanent station assignments at one of the 44 Indianapolis fire houses will be recruited.The initial approach will be through a written announcement from the Chiefs of the Indianapolis Fire Department (IFD)/ Fishers FD and the firefighters' local union presidents (see attached letter) informing the IFD/ Fishers FD of the inauguration of the study and their opportunity to voluntarily participate. Next, human subjects-trained staff from Public Safety Medical (PSM) will visit each fire house to approach IFD/Fishers members for informed consent and determine their eligibility. Their recruitment script is attached. IFD/ Fishers members eligible for study consent and participation will include those: a) permanently assigned to one of the 44 IFD stations or one of 6 Fishers stations; b) with a fire department medical in the last two years; c) at least 18 years of age; and d) full, modified or restricted duty status at randomization; OR e) Firefighters categorized as "Administrative Staff" by the IFD/ Fishers will also be approached for participation. The fire department will notify PSM regarding criteria a, c, d and e and PSM has a record b (medical exams). First, they will provide a brief overview of the study to the firefighters by reiterating the information from the Chief and Union President's letter and the approved informed consent form. They will inform the fire house of its intervention assignment in the study – in other words, whether they will be receiving the nutritional intervention immediately (In Phase 1); or after 12 months (Phase 2). Next, each member will be offered an informed consent form to review on his/her own and will be allowed at least 15 minutes and longer if desired to review it on their own and to discuss it with their colleagues or others. Finally, each member will have a chance to ask the PSM staff questions individually in private areas of the fire house and may decide to sign the form or decline.

IFD/Fishers members will be assured that participation status in the study is completely voluntary and will have no bearing on their employment with the department or on the occupational health care they receive from PSM. Consented participants will be assigned to the Phase 1 nutritional intervention (Group 1) or No intervention (Group 2) according to their fire station's cluster randomization. "Administrative" firefighters who consent will be assigned to Group 1 because they are not assigned to specific fire houses.

For the biomarkers study randomly 100 participants (50 in each group) will be invited to participate in the studyvia email at the same time as the follow-up reminder. . The recruitment will stop when we reach 60 participants (30 in each group)

When: Recruitment will begin as soon as possible following the study's approval by the IRB with the Fire Chief and Union President letter being sent out to all department members. In person recruitment as described above will follow shortly thereafter, on a schedule to visit all 44 IFD / 6 Fishers fire

stations during regular business hours as many times as needed to approach all members with potential interest. For the biomarkers study recruitment will start by the end of December 2017/January 2018. We expect this process to take 4-8 weeks.

Where: The Fire Chief and Union President letter will be sent by mail and email to IFD /Fishers members. The in-person recruitment will take place in each fire station as described above. The PSM researchers will then be visiting each IFD / Fishers fire station to speak with the firefighters about the study and offer them the opportunity to participate. These discussions will take place in common areas of the fire houses such as kitchen/dining area and living rooms, while the individual consenting and question/answer will take place in areas of the fire house that provide privacy.

6.2. Are there any materials that will be used to recruit participants, e.g., emails, posters, and scripts?

☐**No** ☒**Yes: If yes; provide a list of the materials (also include copies with the application)**

The attached Fire Chief and Union President letter with letters from national fire service organizations that endorse our study (IAFF and NFFF) attached, as well as the "Public Safety Medical" script.

7. Available Resources

7.1. Describe the feasibility of recruiting the required number of participants within the recruitment period

The recruitment process is highly feasible. Previously, we have been very successful in participant recruitment from the Indianapolis Fire Department (IFD). In a study of cardiac imaging now completed within the IFD, about 90% of IFD members approached consented and completed the study. We have also worked successfully with Fishers FD in the past on imaging studies Additionally, the proposed research will succeed in large part due to the solid fire service partner commitments. ***Indianapolis Fire Department*** (IFD), Fishers FD and ***Indianapolis Local 416*** support is crucial for recruiting participants and for conducting. Furthermore, the research proposal has been endorsed by significant national fire service partners, which should increase trust and willingness to participate. Moreover, our study designs are such that all firefighters who consent will receive the Mediterranean Diet Nutritional Intervention at some point and have an opportunity for health benefits. Therefore, we expect no problems identifying and recruiting the necessary participants.

7.2. Describe how the Principal Investigator will ensure that a sufficient amount of time will be devoted to conducting and completing the research

This project will be conducted as sponsored research and a grant has been awarded to the PI by the US Department of Homeland Security. The PI is devoting at least 10-15% of his effort to this project through the above award from September 2016 on. The PI and his team will also be continuing weekly meetings with our colleagues in Indianapolis to ensure that the study proceeds as close as possible to its proposed timeline.

7.3. Are there research staff members, in addition to the Principal Investigator?

☐**No: If no, skip to <u>1.a</u>**

☒**Yes: If yes; outline training plans to ensure that research staff members are adequately informed about the protocol and study-related duties**

Yes, this project consists of recruitment and data collection in Indianapolis by Public Safety Medical (PSM) with supervision, direction and data analysis by the Harvard TH Chan School of Public Health team. All Harvard and PSM staff are CITI-certified, have completed institutional HIPPA training and have appropriate training in data protection and study protocols. All staff members will be familiar will this protocol and will be trained in further detail through our weekly meetings and on-site supervision from Dr. Moffatt, the site PI in Indianapolis. All Harvard staff have all been actively participating in this project since its initiation. Therefore, they are all well informed of the details of this project.

7.4. Describe the minimum qualifications for each research role (e.g., RN, social worker) their experience in conducting research, and their knowledge of local study sites and culture

All roles require CITI-certification, completion of institutional HIPPA training and appropriate training in data protection and study protocols.

Overall PI: Dr. Kales- please see sections 3.2 and 7.2 above.

Study site PI: A physician with fire service experience and close relationship to the Indianapolis Fire Department. *Steven Moffatt MD/ Public Safety Medical*- Dr. Moffatt leads an occupational medicine group, Public Safety Medical (PSM), caring for almost 10,000 public safety personnel each year, specializing in preventive care. Dr. Moffatt is a Co-Investigator on several other fire service studies with Dr. Kales. He is very familiar with the local study site and culture, as he has served as the fire department physician for the IFD for some two decades.

Indianapolis Dietician: Should be a Registered Dietitian with working experience with firefighters.

Indianapolis-based research assistants and project coordinator: employed or contracted by Public Safety Medical with sufficient clinical training to collect the assessments described above in section 4.7 above.

Lead Statistician: faculty appointment or equivalent in Biostatistics with experience in related research.

a. Briefly describe how the research facilities and equipment at the research site(s) support the protocol's aims, e.g., private rooms available for interviews, etc.

Harvard and its affiliated facilities provide full managerial, administrative and statistical expertise and software, as well as world-class computational, electronic and physical library facilities to support the proposed research.

In Indianapolis, each fire station has the facilities required to support the protocol's aims. Each fire station has rooms with audiovisuals for the group sessions, full kitchen facilities for cooking demonstrations and regular meal preparation, and private areas for conducting the informed consent process and assessing weights and blood pressures.

b. Are there provisions for medical and/or psychological support resources (e.g., in the event of incidental findings, research-related stress)?
☐ No ☒ Yes: If yes; describe the provisions and their availability

Public Safety Medical may provide psychological services as needed or referrals to other resources in the community in the unlikely event of stress resulting from incidental findings or other research-

related stress.

8. Vulnerable Populations

c. Are there any potentially vulnerable populations (e.g., children, pregnant women, human fetuses, neonates, prisoners, elderly, economically disadvantaged, employees or students of the investigator or sponsor, undocumented, terminally ill, cognitively impaired or mentally ill, etc.)?

☒**No: If no, skip to e**

☐**Yes: If yes; identify all vulnerable populations**

d. Describe safeguards to protect their rights and welfare

N/A.

9. Consent Process

e. Will consent to participate be obtained?

☐**No: If no, skip to i**

☒**Yes: If yes; describe the setting, role of individuals involved, timeframe(s), and steps to minimize coercion/undue influence during the consent process (at the time of initial consent and throughout the study)**

Consent to participate will be obtained prior to any study participation. Human subjects-trained staff from Public Safety Medical (PSM) will visit each fire house to approach eligible IFD / Fishers FD members for informed consent. First, they will provide a brief overview of the study to the firefighters by reiterating the information from the Chief and Union President's letter and the approved informed consent form. IFD /Fishers members will be assured that participation status in the study is completely voluntary and will have no bearing on their employment with the fire department or on the occupational health care they receive from PSM. This assurance is reiterated in both Chief's /Union President's letter and the approved informed consent form. Each member will be offered an informed consent form to review on his/her own. Finally, each member will have a chance to ask the PSM staff questions individually in private areas of the fire house and may decide to sign the form or decline without any pressure from other firefighters. Participants are reminded throughout the study of their rights to withdraw at any time of the study. Any new information which might influence a participant's decision to continue participation will be provided to participants, including re-consent where applicable.

f. Are there any special populations?

☒**No** ☐**Yes: If yes; describe the process to obtain consent, permission or assent**

g. Will consent of the participants be documented in writing?

☒**Yes** ☐**No: If no; describe the rationale for requesting a waiver or alteration of documentation of consent (and/or parental permission)**

h. Will participants be provided with a copy of their signed consent form or information sheet (when a consent form is not signed)?

☒**Yes** ☐**No: If no; explain any extenuating circumstances that make it impossible or inappropriate to meet this requirement, i.e., doing so may place participants at increased risk, if inadvertently disclosed**

i. Is a waiver or alteration of consent (and/or parental permission) being requested?

☒**No** ☐**Yes: If yes; describe the rationale for the request. If the alteration is because of deception or incomplete disclosure, explain whether and how participants will be debriefed**

(include any debriefing materials with the application)

10. Risks

j. Are there any <u>reasonably foreseeable</u> risks, discomforts, and inconveniences to participants and/or groups/communities?

☒No ☐Yes: **If yes; indicate probability, magnitude, and duration of each (note that risks may be physical, psychological, social, legal, and/or economic)**

The only foreseeable risks involve breaches of privacy/confidentiality for most participants. For those in the nested biomarker study, risks associated with blood sample collection may include pain and/or bruising at the needle injection site. Although rare, localized clot formation and infections may occur. Lightheadedness and/or fainting may also occur during or shortly after the blood draw

k. Identify whether any of the information collected, if it were to be disclosed outside of the research, could <u>reasonably</u> place the participant at risk of criminal or civil liability or be damaging to the participant's financial standing, employability or reputation.

None of the information collected, if it were to be disclosed outside of the research, could <u>reasonably</u> place the participant at risk of criminal or civil liability. Furthermore, none of the information collected as part of the research could reasonably be expected to damage any participant's financial standing, employability or reputation.

l. Outline provisions in place to minimize risk

The proposed research presents minimal risk. The Mediterranean diet is proven to be effective and safe for both healthy individuals and those with chronic conditions such as diabetes and cardiovascular disease. The only foreseeable risks involve breaches of privacy/confidentiality, and we have provisions in place to minimize these potential risks. A number of provisions are in place to protect participants' privacy as outlined above in the sections on recruitment, consent and data collection. During the consent process, potential participants will have the right to ask questions and complete forms in private areas. Questionnaire data will be submitted by the participants online via a password-protected site. Height, weight, body fat and blood pressures will be measured privately. At the time of consent, each consented participant will be assigned a unique study ID number. These consent forms and the linkages between the participant names and study ID codes will be kept in separate locked file drawers and in a password-protected computer at the Public Safety Medical (PSM) with access limited to the PSM site PI and PSM project coordinator. Questionnaire data will be submitted to Harvard using Qualtrics by the participants online via a password-protected site. The questionnaire responses will be identified by each participant's unique study ID.Any medical exam or information coming from the biomarkers study as well as the study measures (height, weight, body fat and blood pressures) will be transferred to Harvard in coded form without personal identifiers and using study ID codes in password-protected files. Only approved Harvard research staff will have access to these data sets which will be kept on password- protected computers at Harvard protected by firewalls. Thus, Harvard researchers have access only to de-identified data ensuring that the research has a very low risk of breaching any confidentiality. Any publications resulting from the proposed projects will contain no identifying information associated with participants and will be reported in aggregate.

Urine, blood, and stool samples will be coded. The same procedure is going to take place to the stored samples for future analysis. The key to the code will connect the name with the questionnaires and health information collected throughout the study. The study team will keep the key to the code in a password protected computer and locked file.

11. Benefits

m. Describe potential benefits of study participation (indicate if there is no direct benefit)

Study participation with successful adherence to a Mediterranean diet is expected to provide direct benefits to the participants regarding body composition, glucose and lipid metabolism, the risks of cardiovascular disease and cancer and future risk of cognitive decline.

n. Describe potential benefits of the research to the local community and/or society

If the research is successful in producing behavior change among the firefighters towards a Mediterranean diet, the further dissemination and implementation of such a model nationally in the fire service will be expected to reduce firefighters' cardiovascular morbidity and mortality; obesity-related costs on injuries, workers compensation and disability; and costs related to other chronic health conditions; as well as decrease cancer risks.

These changes would be beneficial to society. Healthier firefighters would be expected to perform better. Moreover, more than 35 states have "presumptive" laws entitling firefighters with heart disease to receive publicly funded disability and/or death benefits. Obesity is also very costly as an independent risk for workers' compensation injuries and disability among firefighters. As a result, there are many direct and indirect costs involved totaling billions of dollars around the country. These include: lost work hours, higher insurance premiums, overtime, disability and early retirement payments.

12. Reportable Events

o. Outline plans for communicating reportable events (e.g., adverse events, unanticipated problems involving risks to participants or others, breach of confidentiality)

We plan to notify the Harvard IRB of any reportable events within 5 business days of becoming aware of any such event.

13. Research Related Injuries (this section must be completed for any greater than minimal risk research)

13.1 Are there provisions for medical care and compensation for research-related injuries?

☒No ☐Yes: **If yes; outline these provisions (Please note that although Harvard's policy is not to provide compensation for physical injuries that result from study participation, medical treatment should be available including first aid, emergency treatment and follow-up care as needed. If the research plan deviates from this policy, provide appropriate justification.)**

N/A. Minimal risk.

14. Participant Privacy

p. Describe provisions to protect participants' privacy (their desire to control access of others to themselves, e.g., the use of a private interview room) and to minimize any sense of intrusiveness that may be caused by study questions or procedures

A number of provisions are in place to protect participants' privacy as outlined above in the sections on recruitment, consent and data collection. During the consent process, potential participants will have the right to ask questions and complete forms in private areas. Questionnaire data will be submitted by the participants online via a password-protected site. Height, weight, body fat and blood

pressures will be measured privately.

15. Data Confidentiality

q. Will the information that is obtained be recorded in such a manner that participants can be identified, directly or through identifiers linked to the participants?

☐**No: If no, skip to u**

☒**Yes: If yes; either state that participants will be told that their data will be public or describe provisions to maintain the confidentiality of identifiable data, e.g., use of password protections (please refer to the Harvard Research Data Security Policy (HRDSP), at http://vpr.harvard.edu/pages/harvard-research-data-security-policy, for additional information about required data security measures) [NOTE: The HRDSP does not always apply if data are not being stored at Harvard facilities. Please consult the HRDSP for additional information.]**

At the time of consent, each consented participant will be assigned a unique study ID number. These consent forms and the linkages between the participant names and study ID codes will be kept in separate locked file drawers and in a password-protected computer at the Public Safety Medical (PSM) office with access limited to the PSM site PI and PSM project coordinator.

Questionnaire data will be submitted to Harvard using Qualtrics by the participants online via a password-protected site. The questionnaire responses will be identified by each participant's unique study ID.

Any medical exam and study measures (height, weight, body fat and blood pressures) will be transferred to Harvard in coded form without personal identifiers and using study ID codes in password-protected files. Only approved Harvard research staff will have access to these data sets which will be kept on password-protected computers at Harvard protected by firewalls. Thus, Harvard researchers have access only to de-identified data ensuring that the research has a very low risk of breaching any confidentiality.

Any publications resulting from the proposed projects will contain no identifying information associated with participants and will be reported in aggregate.

r. Describe i) whether data will be transmitted, and if so how; ii) how long it will be stored; and iii) plans for the data at the end of the storage period (how will it be destroyed, or will it be returned to data provider)

Data will only be transmitted in coded format. Please see the above section regarding data transmittals to Harvard research staff. Prior to transferring data to the study statistician, data advisory board or other colleagues, a new (second) code will be added to each participant's data and the original study ID code will be removed by Harvard staff. The linkages between these codes will be will be kept in separate locked file drawers and on a password-protected computer at Harvard with access limited to the Harvard PI and Harvard project coordinator.

After study is completed the research data will be stored on password-protected computers in locked rooms for at least 7 years after study closure per OHRA's Record Retention Policy.

s. Indicate how research team members and/or other collaborators are permitted access to information about study participants

Analyses and any access by other collaborators (Tufts co-investigators on the grant) will only use the de-identified data sets in password-protected files with the second set of study codes that are not directly linked to the participants' identifiers. Thus, further minimizing the already low risk if breaching confidentiality.

t. If future use of data, data sharing, i.e., required of NIH-funded studies using/generating large-scale human genomic data, or future open access, i.e., free availability and unrestricted use, of data is planned or likely, indicate how data will be shared/released.

N/A

16. Costs and Payments

u. Identify any costs that participants may incur during the study, including transportation costs, childcare, or other out-of-pocket expenses

No costs.

v. Is there any payment or reimbursement that participants may receive during the study?

No ☒ ☐Yes: If yes; specify the amount, method and timing of disbursement. (Please refer to Harvard University Financial Policy on Human Subject Payments at http://policies.fad.harvard.edu/pages/human-subject-payments)

For the biomarkers study, participants will be compensated with $100 at the first visit and a $100 gift card at the second visit.

17. Multi-site Study Management

w. Is this a multi-site study?

☒No ☐Yes: If yes; describe plans for communication among sites regarding adverse events, interim results, protocol modifications, monitoring of data, etc.

18. Investigational Drug/Biologic/Device

x. Does this study involve an Investigational Drug/Biologic/Device?

☒No: If no; skip to cc

☐Yes: If yes; identify and describe the drug/biologic/device (e.g., marketing status: Is there an IND/IDE, classification of a device as significant vs. non-significant risk)

y. Describe its administration or use

z. Compare the research drug/biologic/device to the local standard of care

aa. Describe plans for receiving, storage, dispensing and return (to ensure that they will be used only for participants and only by authorized investigators)

bb. If proven beneficial, describe anticipated availability and cost to participants post-study; plans (if applicable) to make available

19. HIPAA Privacy Protections

cc. Are HIPAA privacy protections required? Please note that only Harvard University Health Services and Harvard School of Dental Medicine are covered entities at Harvard. Harvard is

otherwise not a HIPAA covered entity. If, however, data is derived from a Covered Entity (e.g. a hospital or community health center), mark 'yes' and address the items below.

☐ **No: If no; skip to dd**

☒ **Yes: If yes; include at least one of the following:**

Describe plans for obtaining authorization to access protected health information

Harvard team will access coded clinical data from consented subjects **ONLY**, derived from the annual fire department (not research-related) medical examinations conducted by Public Safety Medical. These data will include Protected Health Information. The release of this

information is addressed through the use of the appropriate consent form for HIPAA covered entities which provides authorization to use and/or share Protected Health Information (PHI).

Provide the rationale for a waiver of authorization or limited waiver of authorization request

20. Data and Specimen Banking

dd. Does the study include Data and Specimen Banking?

☒ **Yes: If yes; identify what will be collected and stored, and what information will be**
☐ **associated with the specimens**
No: If no; skip to hh

As previously mentioned for the biomarkers study we will be banking blood, serum samples and stools samples at Ohio University. The samples will be de-identified and coded with the unique ID that connects the participant with the questionnaires and health information from the previously mentioned medical exams (at rest and maximal heart rate, systolic and diastolic blood pressure, heart rate recovery, electrocardiographic findings at rest, hip and waist circumference, body composition, data regarding the grip strength, leg-press and push-ups, and routine lab values (e.g. lipid panels, hs-CRP and glucose)).

ee. Describe where and how long the data/specimens will be stored and whether participants' permission will be obtained to use the data/specimens in other future research projects

For the biomarkers study, the samples will be de-identified and coded, initially processed at Clinical and Translational Support (CTSL), Indianapolis. The remaining aliquots not use directly for the study will be storage at Ohio University for at least two years. Participants' permission will be obtained to use the data/specimens in other future research projects

ff. Identify who may access data/specimens and how

Only approved Harvard research staff and collaborators will have access to the data/specimens which will be storage at OU. Thus, the approved researchers have access only to de-identified samples and coded data ensuring that the research has a very low risk of breaching any confidentiality. Any publications resulting from the proposed projects will contain no identifying information associated with participants and will be reported in aggregate. The key to the code will connect the name with the questionnaires and health information collected throughout the study. The study team will keep the key to the code in a password protected computer and locked file.

gg. Will specimens and/or data be sent to research collaborators outside of Harvard?

☐ **No** ☒ **Yes: If yes; describe the plan**

The urine specimens will be sent for analyses by Dr. Magiatis. When the data is sent to research collaborators outside Harvard, as previously described, it will be de-identified data ensuring that the research has a very low risk of breaching any confidentiality

Please see reply 9.c. above.

20.5 Will specimens and/or data be received from collaborators outside of Harvard?
☐No ☒Yes: **If yes; describe the plan**

Please see descriptions above to received coded data from Public Safety Medical, from Ohio University and University of Athens.

21. Sharing Study Results

hh. Is there a plan to share study results with individual participants?
☐No ☒Yes: **If yes; describe the plan**

We plan to share only weight, anthropometric measures and blood pressure at the time of their measurement with each participant. As these will be measured in private, they will be shared verbally in private (see above)

ii. Is there a plan to disseminate aggregate results to the community where the research is conducted?
☐No ☒Yes: **If yes; describe the plan**

At the end of the study we plan to make a report to share with the IFD/ Fishers FD, their family members and the US fire service in general. Aggregate/De-identified data will be shared with the general public through community presentations and in the media.

22. Regulatory Compliance

j. Describe plan for monitoring regulatory compliance, in order to ensure proper record keeping and retention of required regulatory documents

The PI will immediately upon knowledge (1) notify the IRB of any protocol changes prior to starting or progressing with the study, (2) report to the IRB any information related to non-compliance with federal regulations pertaining to research or IRB requirements or determinations, and (3) responds to all requests from the IRB for further information or clarification regarding concerns or issues related to this study. All IRB documents will be maintained in ESTR and regulatory documents maintained in a regulatory binder which will be stored in a locked area and maintained for 7 years after study closure per OHRA's Record Retention policy.
