# Supplementary material for: Effect of a Nutrition Intervention on Mediterranean Diet Adherence Among Firefighters: A Cluster Randomized Clinical Trial
Source: JAMA Netw Open. 2023 Aug 17;6(8):e2329147. doi: 10.1001/jamanetworkopen.2023.29147 (PMC10436136; doi:10.1001/jamanetworkopen.2023.29147)
Supplement: Supplement 2. — eTable 1. Baseline Characteristics of Feeding America’s Bravest Participants by Follow-Up Completion eTable 2. Multiple Imputation Between-Group Differences for 6- and 12-Month Changes in mMDS Items eTable 3. Between-Group Differences for 6- and 12-Month Changes in Cardiometabolic Parameters and 95% CIs Corrected for Multiple Comparisons eTable 4. Multiple Imputation Between-Group Differences for 6- and 12-Month Changes in Cardiometabolic Parameters eTable 5. Description of the Modified Mediterranean Diet Scoring System eFigure 1. Influence Analysis Shows the Influence of Each Imputation Model Estimate on the Overall Effect Size Estimates for A) 6-Month and B) 12-Month Between-Group Differences in mMDS Changes eFigure 2. Quality Check for Multilevel Multiple Imputation Model #1; Histograms Show a Modified Mediterranean Diet Score (0-51 Points) for the Completed vs Observed Datasets and Mean (SD) by Study Group eFigure 3. Quality Check for Multilevel Multiple Imputation Model #1; Kernel Density Plots Show Observed, Completed, and Imputed Modified Mediterranean Diet Score Values for 6- and 12-Month Follow-Up eTable 6. Quality Check for Multilevel Multiple Imputation Model #1; Summary Statistics Compare Observations With Completed Dataset Values for the Modified Mediterranean Diet Score at 6- and 12-Month Follow-Up eMethods. Original Study Design and Intervention [file jamanetwopen-e2329147-s002.pdf]

Supplemental Online Content

Hershey MS, Chang CR, Sotos-Prieto M, et al. Effect of a nutrition intervention on Mediterranean diet adherence among firefighters: a cluster randomized clinical trial. *JAMA Netw Open*. 2023;6(8):e2329147. doi:10.1001/jamanetworkopen.2023.29147

eTable 1. Baseline Characteristics of Feeding America's Bravest Participants by Follow-Up Completion

eTable 2. Multiple Imputation Between-Group Differences for 6- and 12-Month Changes in mMDS Items

eTable 3. Between-Group Differences for 6- and 12-Month Changes in Cardiometabolic Parameters and 95% CIs Corrected for Multiple Comparisons

eTable 4. Multiple Imputation Between-Group Differences for 6- and 12-Month Changes in Cardiometabolic Parameters

eTable 5. Description of the Modified Mediterranean Diet Scoring System

eFigure 1. Influence Analysis Shows the Influence of Each Imputation Model Estimate on the Overall Effect Size Estimates for A) 6-Month and B) 12-Month Between-Group Differences in mMDS Changes

eFigure 2. Quality Check for Multilevel Multiple Imputation Model #1; Histograms Show a Modified Mediterranean Diet Score (0-51 Points) for the Completed vs Observed Datasets and Mean (SD) by Study Group

eFigure 3. Quality Check for Multilevel Multiple Imputation Model #1; Kernel Density Plots Show Observed, Completed, and Imputed Modified Mediterranean Diet Score Values for 6- and 12-Month Follow-Up

eTable 6. Quality Check for Multilevel Multiple Imputation Model #1; Summary Statistics Compare Observations With Completed Dataset Values for the Modified Mediterranean Diet Score at 6- and 12-Month Follow-Up

eMethods. Original Study Design and Intervention

This supplementary material has been provided by the authors to give readers additional information about their work.

© 2023 Hershey MS et al. *JAMA Network Open*.

eTable 1. Baseline Characteristics of Feeding America's Bravest Participants by Follow-Up Completion

	**Complete follow-up (n=260)**	**Incomplete 1 yr. follow-up (n=225)**
Study group (%)		
Control	61.54	37.50
Intervention	38.46	62.50
Indianapolis Fire Department (%)	87	88
Fishers Fire Department (%)	13	12
mMDS (points)	23.74 (6.65)	22.45 (6.41)
Age (yrs)	45.62 (7.75)	45.82 (8.61)
Sex (%)		
Male	96.15	92.41
Female	3.10	7.75
Race (%)		
Caucasian	83.08	82.59
African American	13.46	15.18
Other; Asian, native Hawaiian or Pacific Islander, and American Indian or Alaska native	3.46	2.23
Marital status		
Married	82.00	77.69
Single; divorced, widowed, or never married	18.00	22.31
BMI (kg/m^2)	29.86 (4.28)	30.23 (4.56)
Percent body fat (%)	27.54 (6.49)	28.83 (6.51)
Waist circumference (cm)	99.49 (11.99)	99.98 (13.18)
Glucose (mg/dl)[a]	99.70 (20.98)	99.19 (17.44)
Total cholesterol (mg/dl)[a]	195.30 (37.85)	198.40 (36.96)
HDL cholesterol	47.54 (11.00)	49.81 (11.62)
LDL cholesterol	122.71 (33.06)	123.75 (31.75)
Triglycerides (mg/dl)[a]	124.85 (70.88)	125.89 (81.76)
Education level (%)		
Technical school/some college/associate degree	64.68	70.25
Bachelor's degree or higher	35.32	29.75
Total energy intake (kcal/d)	2531 (1083)	2267 (1140)
Smoking status (%)		
Never	61.69	61.16
Current	7.46	12.40
Former	30.85	26.45
Physical activity (%)		
No regular exercise[b]	10.77	15.97
Regular modest exercise[c]	20.00	23.53
Regular heavy exercise[d]	69.23	60.50
Chronic conditions[e] (%)		
No	67.05	73.42
Yes	32.95	26.58

d:day, dL: deciliter, g: grams, hrs: hours, , in: inches, HDL: high-density lipoprotein, kcal; kilocalories, kg: kilogram, LDL: low density lipoprotein, mg: milligram, m: meters, min: minutes, mL: milliliters, mMDS: modified Mediterranean diet score, wk: week, yrs: years. Values are means (SD) for continuous variables and % for qualitative variables.

[a]Glucose and lipid profiles were determined using fasting blood samples collected during fire department medical examinations.

[b]Did not participate regularly in programmed recreation, sport, or heavy physical activity; avoids walking or exertion.

[c]Participated regularly in recreation or work requiring modest physical activity, such as golf, horseback riding, calisthenics, gymnastics, table tennis, bowling, weightlifting, yard work; walks for pleasure, routinely use stairs.

[d]Participated regularly in heavy physical exercise such as running or jogging, swimming, cycling, rowing, skipping rope, running in place, or engaging in vigorous aerobic activity such as tennis, basketball, or handball; 10-60 minutes per week.

[e]Defined as self-reported cardiovascular disease, hypertension, arrythmia, diabetes, dyslipidemia, or angina.

© 2023 Hershey MS et al. *JAMA Network Open*.

eTable 2. Multiple Imputation Between-Group Differences for 6- and 12-Month Changes in mMDS Items

mMDS items	**multiple imputation[a] mean points (95%CI)[b]**		
	Control	Mediterranean Diet Nutrition Intervention	Between-group differences[b]
**1. Fast-food or Take-out food (0-4 pts)**			
6-month change	***0.40 (0.23 to 0.57)***	***0.34 (0.18 to 0.51)***	-0.06 (-0.29 to 0.18)
12-month change	***0.49 (0.33 to 0.65)***	***0.49 (0.33 to 0.64)***	-0.004 (-0.22 to 0.21)
**2. Fruits (0-4 pts)**			
6-month change	-0.004 (-0.14 to 0.13)	0.10 (-0.06 to 0.26)	0.10 (-0.11 to 0.31)
12-month change	-0.03 (-0.17 to 0.11)	0.11 (-0.05 to 0.27)	0.14 (-0.07 to 0.35)
**3. Vegetables (not including potatoes) (0-4 pts)**			
6-month change	***-0.25 (-0.44 to -0.06)***	*0.04 (-0.11 to 0.20)*	***0.29 (0.05 to 0.54)***
12-month change	***-0.15 (-0.30 to -0.01)***	0.03 (-0.17 to 0.22)	0.18 (-0.06 to 0.42)
**4. Sweet Desserts (e.g. cake, cookies, pie, ice cream) (0-4 pts)**			
6-month change	***0.74 (0.46 to 1.02)***	***0.58 (0.32 to 0.83)***	-0.16 (-0.54 to 0.21)
12-month change	***0.93 (0.66 to 1.20)***	***0.83 (0.55 to 1.11)***	-0.10 (-0.47 to 0.27)
**5. Primary cooking oil/fat use at home (0-5 pts)**			
6-month change	***0.43 (0.12 to 0.75)***	***0.57 (0.24 to 0.91)***	0.14 (-0.32 to 0.59)
12-month change	***0.36 (0.02 to 0.70)***	***0.56 (0.15 to 0.98)***	0.20 (-0.33 to 0.74)
**6. Primary cooking oil/fat use at work (0-5 pts)**			
6-month change	***0.48 (0.21 to 0.75)***	***0.69 (0.30 to 1.07)***	0.21 (-0.27 to 0.69)
12-month change	***0.31 (0.01 to 0.61)***	***0.72 (0.31 to 1.13)***	0.41 (-0.10 to 0.91)
**7. Fried foods (e.g. French fries, fried chicken, nuggets) (0-4 pts)**			
6-month change	***0.55 (0.33 to 0.77)***	***0.39 (0.20 to 0.59)***	-0.15 (-0.45 to 0.14)
12-month change	***0.57 (0.37 to 0.76)***	***0.48 (0.27 to 0.69)***	-0.09 (-0.38 to 0.21)
**8. Breads/starches consumed at home (0-4 pts)**			
6-month change	***0.57 (0.30 to 0.83)***	***0.64 (0.32 to 0.96)***	0.08 (-0.33 to 0.48)
12-month change	***0.69 (0.42 to 0.97)***	***0.68 (0.27 to 1.09)***	-0.01 (-0.44 to 0.42)
**9. Breads/starches consumed at work (0-4 pts)**			
6-month change	***0.35 (0.04 to 0.66)***	***0.75 (0.41 to 1.08)***	0.40 (-0.05 to 0.84)
12-month change	***0.58 (0.19 to 0.97)***	***1.00 (0.57 to 1.43)***	*0.42 (-0.12 to 0.96)*
**10. Baked, broiled, grilled, or blackened ocean fish (0-4 pts)**			
6-month change	***-0.22 (-0.36 to -0.07)***	-0.06 (-0.20 to 0.07)	0.15 (-0.04 to 0.34)
12-month change	***-0.27 (-0.41 to -0.12)***	-0.01 (-0.18 to 0.15)	***0.25 (0.03 to 0.47)***
**11. Quantity of alcoholic beverages (0-4 pts)**			
6-month change	0.01 (-0.21 to 0.24)	-0.07 (-0.31 to 0.18)	-0.08 (-0.41 to 0.26)
12-month change	-0.08 (-0.31 to 0.15)	-0.21 (-0.45 to 0.04)	-0.13 (-0.45 to 0.20)
**12. Wine consumption (0-2 pts)**			
6-month change	***-0.16 (-0.28 to -0.05)***	-0.11 (-0.23 to 0.01)	0.05 (-0.11 to 0.22)
12-month change	***-0.19 (-0.30 to -0.08)***	***-0.15 (-0.27 to -0.02)***	0.05 (-0.12 to 0.21)
**13. Non-alcoholic beverages at home (0-4 pts)**			
6-month change	***-0.40 (-0.63 to -0.16)***	-0.04 (-0.31 to 0.23)	***0.36 (0.00 to 0.71)***
12-month change	***-0.45 (-0.80 to -0.10)***	-0.13 (-0.48 to 0.23)	0.32 (-0.17 to 0.81)
**14. Non-alcoholic beverages at work (0-4 pts)**			
6-month change	-0.21 (-0.48 to 0.07)	-0.05 (-0.32 to 0.22)	0.16 (-0.23 to 0.54)
12-month change	***-0.38 (-0.67 to -0.10)***	-0.30 (-0.68 to 0.07)	0.08 (-0.40 to 0.56)
**15. Legumes (e.g. beans, chickpeas, lentils) (0-4 pts)**			
6-months	-0.10 (-0.26 to 0.07)	0.06 (-0.09 to 0.21)	0.15 (-0.07 to 0.38)
12-months	***-0.17 (-0.31 to -0.02)***	0.08 (-0.07 to 0.23)	***0.25 (0.03 to 0.46)***
**16. Nuts (e.g. walnuts, almonds, hazelnuts, pistachio, peanuts) (0-4 pts)**			
6-month change	-0.14 (-0.36 to 0.09)	0.02 (-0.21 to 0.25)	0.16 (-0.15 to 0.47)
12-month change	-0.15 (-0.36 to 0.07)	0.08 (-0.16 to 0.31)	0.23 (-0.09 to 0.54)

[a]Multiple imputation results are pooled estimates from each of the 25 completed datasets comprised of observed and imputed values.
[b]Multilevel mixed-effects linear regressions for repeated measures (baseline, 6-month, and 12-month study visits) and study group (intervention vs control) on mMDS score changes at 6- and 12-month follow-up and between group differences are adjusted for age, sex, fire department, physical activity, race, and waist circumference. Fire stations (52 clusters) and participants (n=484) were specified as the random effects parameters.

© 2023 Hershey MS et al. *JAMA Network Open*.

eTable 3. Between-Group Differences for 6- and 12-Month Changes in Cardiometabolic Parameters and 95% CIs Corrected for Multiple Comparisons

Cardiometabolic parameters		**6-month mean points (95%CI)[a]**			**12-month mean points (95%CI)[a]**		
		6-month change estimate	6-month between-group differences (nominal 95%CI)	adjusted 95%CI[c]	12-month change	12-month between-group differences (nominal 95%CI)	adjusted 95%CI[c]
**BMI**			-0.20 (-0.57 to 0.18)	(0.52 to -0.92)		-0.13 (-0.48 to 0.22)	(0.66 to -0.92)
	control	***0.60 (0.29 to 0.90)***			***-0.26 (-0.50 to -0.01)***		
	intervention	***-0.40 (0.18 to 0.61)***			***-0.38 (-0.64 to -0.13)***		
**Body fat percentage**			***-0.79 (-1.40 to -0.18)***	(0.31 to -1.89)		-0.59 (-1.25 to 0.08)	(0.87 to -2.05)
	control	***0.87 (0.42 to 1.31)***			-0.24 (-0.70 to 0.23)		
	intervention	0.08 (-0.37 to 0.53)			***-0.82 (-1.29 to -0.36)***		
**Waist circumference**			0.90 (-0.70 to 2.50)	(-2.35 to 4.15)		-0.10 (-1.66 to 1.47)	(1.46 to -1.66)
	control	***-2.42 (-3.51 to -1.33)***			***1.24 (0.31 to 2.16)***		
	intervention	***-1.52 (-2.70 to -0.34)***			1.14 (-0.16 to 2.43)		
**Total cholesterol[b]**			-4.57 (-13.67 to 4.54)	(11.91 to -21.05)		3.16 (-7.58 to 13.90)	(-16.01 to 22.33)
	control	2.58 (-3.23 to 8.40)			-1.47 (-9.88 to 6.93)		
	intervention	-1.98 (-9.04 to 5.08)			1.69 (-5.10 to 8.47)		
**HDL cholesterol[b]**			2.13 (-0.54 to 4.79)	(-3.78 to 8.04)		1.26 (-0.81 to 3.34)	(-3.28 to 5.80)
	control	-1.83 (-3.57 to -0.08)			-0.70 (-2.11 to 0.71)		
	intervention	0.30 (-1.68 to 2.29)			0.56 (-0.99 to 2.11)		
**LDL cholesterol[b]**			***-6.92 (-13.58 to -0.27)***	(6.72 to -20.56)		1.80 (-7.53 to 11.12)	(-10.16 to 13.76)
	control	3.05 (-1.17 to 7.27)			-0.28 (-6.87 to 6.32)		
	intervention	***-3.88 (-8.93 to 1.17)***			1.52 (-5.11 to 8.15)		
**Glucose[b]**			1.54 (-3.64 to 6.72)	(-7.80 to 10.88)		3.45 (-3.13 to 10.03)	(-8.99 to 15.89)
	control	-0.89 (-3.07 to 1.29)			-0.17 (-3.04 to 2.69)		
	intervention	0.65 (-4.49 to 5.79)			3.28 (-2.51 to 9.07)		
**Triglycerides[b]**			-5.96 (-38.03 to 26.11)	(33.64 to -45.56)		-6.66 (-38.37 to 25.04)	(37.59 to -50.91)
	control	11.24 (-18.42 to 40.89)			3.65 (-21.05 to 28.35)		
	intervention	5.28 (-7.24 to 17.80)			-3.01 (-23.19 to 17.16)		

CI: confidence intervals, BMI: body mass index, SD: standard deviation, HDL: high density lipoprotein, LDL: low density lipoprotein

[a]Multilevel mixed-effects linear regressions for repeated measures (baseline, 6-month, and 12-month study visits) and study group (intervention vs control) on mMDS score changes at 6- and 12-month follow-up and between group differences are adjusted for age, sex, fire department, physical activity, race, and waist circumference, with the exception of waist circumference as the outcome. Fire stations (52 clusters) and participants (n=484) were specified as the random effects parameters.

[b]Glucose and lipid profiles were determined using fasting blood samples collected during fire department medical examinations.

[c]Corrected for multiple comparisons false discovery rate according to Simes multiple-test procedure are based on the sixteen tests performed.

© 2023 Hershey MS et al. *JAMA Network Open*.

eTable 4. Multiple Imputation Between-Group Differences for 6- and 12-Month Changes in Cardiometabolic Parameters

Cardiometabolic parameters		**multiple imputation[a] mean points (95%CI)[b]**			
		6-month change	6-month between-group differences	12-month change	12-month between-group differences
**BMI**			-0.37 (-0.83 to 0.09)		-0.17 (-0.65 to 0.31)
	control	***0.56 (0.20 to 0.91)***		-0.02 (-0.35 to 0.31)	
	intervention	0.19 (-0.12 to 0.50)		-0.19 (-0.58 to 0.20)	
**Body fat percentage**			***-1.03 (-1.82 to -0.23)***		-0.83 (-1.69 to 0.04)
	control	***1.00 (0.40 to 1.59)***		0.43 (-0.14 to 1.00)	
	intervention	-0.03 (-0.59 to 0.53)		-0.40 (-1.05 to 0.24)	
**Waist circumference**			1.31 (-0.84 to 3.46)		-0.54 (-2.78 to 1.70)
	control	***-1.97 (-3.36 to -0.59)***		-0.02 (-1.59 to 1.54)	
	intervention	-0.66 (-2.36 to 1.03)		-0.56 (-2.28 to 1.16)	
**Total cholesterol[c]**			-1.75 (-7.82 to 4.33)		3.38 (-4.44 to 11.20)
	control	0.11 (-3.70 to 3.92)		-3.46 (-8.76 to 1.83)	
	intervention	-1.64 (-6.39 to 3.11)		-0.09 (-6.15 to 5.97)	
**HDL cholesterol[c]**			0.66 (-1.97 to 3.29)		0.25 (-2.55 to 3.06)
	control	-0.96 (-2.97 to 1.04)		-0.65 (-2.50 to 1.21)	
	intervention	-0.30 (-2.22 to 1.61)		-0.40 (-2.55 to 1.75)	
**LDL cholesterol[c]**			-4.19 (-10.27 to 1.89)		2.83 (-3.83 to 9.48)
	control	0.30 (-2.99 to 3.58)		-2.82 (-7.10 to 1.47)	
	intervention	-3.90 (-8.85 to 1.06)		0.00 (-5.31 to 5.32)	
**Glucose[c]**			-1.00 (-6.50 to 4.51)		-1.39 (-7.49 to 4.71)
	control	1.45 (-3.16 to 6.07)		1.51 (-3.04 to 6.05)	
	intervention	0.46 (-2.63 to 3.54)		0.12 (-4.32 to 4.55)	
**Triglycerides[c]**			14.96 (-22.72 to 52.64)		3.35 (-20.81 to 27.51)
	control	5.05 (-11.22 to 22.33)		0.38 (-15.57 to 16.34)	
	intervention	20.01 (-12.74 to 52.77)		3.74 (-15.12 to 22.60)	

CI: confidence intervals, BMI: body mass index, SD: standard deviation, HDL: high density lipoprotein, LDL: low density lipoprotein

[a]Multiple imputation results are pooled estimates from each of the 25 completed datasets comprised of observed and imputed values.

[b]Multilevel mixed-effects linear regressions for repeated measures (baseline, 6-month, and 12-month study visits) and study group (intervention vs control) on mMDS score changes at 6- and 12-month follow-up and between group differences are adjusted for age, sex, fire department, physical activity, race, and waist circumference, with the exception of waist circumference as the outcome. Fire stations (52 clusters) and participants (n=484) were specified as the random effects parameters.

[c]Glucose and lipid profiles were determined using fasting blood samples collected during fire department medical examinations.

© 2023 Hershey MS et al. *JAMA Network Open*.

eTable 5. Description of the Modified Mediterranean Diet Scoring System

Questions were extracted from *Feeding America's Bravest* Qualtrics study surveys on diet and lifestyle, and, if unavailable, from the food frequency questionnaire. The possible lifestyle survey responses (top row) and FFQ responses (bottom row) are matched with their corresponding points (middle row).

Calculation of the mMDS:

mMDS1+(mMDS2+mMDS3)+mMDS4+mMDS7+mMDS10+mMDS12+mMDS15+mMDS16+mMDS11+(mMDS5+mMDS8+mMDS13)*(1-fh)+(mMDS6+mMDS9+mMDS14)*fh

mMDS items	Components	Score range	Points for each answer								
1. Fast-food or Take-out food	How many times per week do you eat the following?	Qual:	never	≤ 1	2-3	3-4	5-6	every day	8-10	≥ 11	-
		0-4	4 pts	3 pts	2 pts		1 pts		0 pts		
		FFQ:	0	0<x<1.5	1.5≤x4.5		4.5≤x<7.5		≤ 7.5		missing
2. Fruits	How many servings of each of the following do you consume per day?	Qual:	0	≤ 1	2-3	3-4	5-6	≥ 7	-		
		0-4	0 pts	1 pts	2 pts	3 pts	4 pts		0 pts		
		FFQ:	0	0<1x<1.5	1.5≤x<3	3≤x<4.5	≥4.5		missing		
3. Vegetables (not including potatoes)	How many servings of each of the following do you consume per day?	Qual:	0	≤ 1	2-3	3-4	5-6	≥ 7	-		
		0-4	0 pts	1 pts	2 pts	3 pts	4 pts		0 pts		
		FFQ:	0	0<1.5	1.5≤x<3	3≤x<4.5	≥4.5		missing		
4. Sweet Desserts (cake, cookies, pie, ice cream, etc.)	How many times per week do you eat the following?	Qual:	never	≤ 1	2-3	3-4	5-6	≥ 7	-		
		0-4	4 pts		3 pts	2 pts	1 pts	0 pts			
		FFQ:	<1.5		1.5≤x<3	3≤x4.5	4.5≤x<6.5	≥ 6.5	missing		
5. Primary cooking oil/fat use at home[a]	Which oil or fat do you use most often for cooking and serving food at home?	Qual:	Butter	Lard or other animal fat	Margarine	Corn or vegetable oil	Benechol or Smart Balance	Olive oil	EVOO	other	-
		0-5	0 pts		1 pts	2 pts	3 pts	4 pts	5 pts	0 pts	
		FFQ:	Butter	Lard	Margarine	Vegetable oil	-	Olive oil	-	missing	
6. Primary cooking oil/fat use at work[b]	Which oil or fat do you use most often for cooking and serving food at the firehouse?	Qual:	Butter	Lard or other animal fat	Margarine	Corn or vegetable oil	Benechol or Smart Balance	Olive oil	EVOO	other	-
		0-5	0 pts		1 pts	2 pts	3 pts	4 pts	5 pts	0 pts	

© 2023 Hershey MS et al. *JAMA Network Open*.

		FFQ:	Butter	Lard	Margarine	Vegetable oil	-	Olive oil	-	missing						
7. Fried foods (French fries, fried chicken, chicken nuggets, etc.)	How many times per week do you eat the following?	Qual:	never	≤ 1	2-3	3-4	5-6	every day	8-10	≥ 11	-					
		0-4	4 pts	3 pts	2 pts	1 pts	0 pts									
		FFQ:	0	0≤x<1.5	1.5≤x<3	3≤x<4.5	≥ 4.5	missing								
8. Breads/starches consumed at home[a]	Which bread or starch do you most frequently eat at home?	Qual:	I do not eat bread or starch	Durum wheat bread or dry pasta	White bread, filled pasta, white rice, or potatoes	French bread or Italian bread or multigrain or other crusty bread	Whole wheat bread or brown rice or whole wheat pasta	-								
		0-4	3 pts		0 pts	2 pts	4 pts	0 pts								
		FFQ:	Whole grains ≥ refined grains		Refined grains > whole grains	-	-	missing								
9. Breads/starches consumed at work[b]	Which bread or starch do you most frequently eat at the firehouse?	Qual:	I do not eat bread or starch	Durum wheat bread or dry pasta	White bread, filled pasta, white rice, or potatoes	French bread or Italian bread or multigrain or other crusty bread	Whole wheat bread or brown rice or whole wheat pasta	-								
		0-4	3 pts		0 pts	2 pts	4 pts	0 pts								
		FFQ:	Whole grains ≥ refined grains		Refined grains > whole grains	-	-	missing								
10. Baked, broiled, grilled, or blackened (NOT fried) ocean fish (salmon, tuna, cod, haddock, etc.)	How many times per week do you eat the following?	Qual:	never	≤ 1	2-3	3-4	5-6	every day	8-10	≥ 11	-					
		0-4	0 pts	1 pts	2 pts	3 pts	4 pts	0 pts								
		FFQ:	0	0≤x<1.5	1.5≤x<3	3≤x<4.5	≥ 4.5	missing								
11. Quantity of alcoholic beverages	How many alcoholic beverages (beer, wine, hard liquor, etc.) do you drink over a typical week?	Qual:	I do not drink	0	1-2	3-4	5-6	7-8	9-10	11-12	13-14	15-16	17-18	19-20	≥ 21	-
		0-4	0 pts	2 pts	4 pts	1 pts	0 pts									
		FFQ:	0≤x<1	1≤x<4.5	4.5≤x<20.5	≥20.5	missing									
12. Wine consumption	When you drink alcoholic beverages, what type do you drink?	Qual:	White wine	Red wine	Beer	Hard liquors	Don't drink									
		0-2	2 pts	0 pts												
		FFQ:	Red/white wine ≥ beer/light beer/liquor	Red/white wine < beer/light beer/liquor												

© 2023 Hershey MS et al. *JAMA Network Open*.

13. Non-alcoholic beverages at home[a]	Which of the following non-alcoholic beverages do you most frequently drink at home?	Qual:	Cola/ soda	Diet cola/soda	Fruit drink or punch	Milk	Juice	Tea/ coffee	Water	Other	-
		0-4	0 pts	1 pts			2 pts		4 pts	0 pts	
		FFQ:	Coke/ soda	Low-calorie beverage	Sugary beverages	Milk	Juice	Tea/ coffee	Water	-	missing
14. Non-alcoholic beverages at work[b]	Which of the following non-alcoholic beverages do you most frequently drink at the firehouse?	Qual:	Cola/ soda	Diet cola/soda	Fruit drink or punch	Milk	Juice	Tea/ coffee	Water	Other	-
		0-4	0 pts	1 pts			2 pts		4 pts	0 pts	
		FFQ:	Coke/ soda	Low-calorie beverage	Sugary beverages	Milk	Juice	Tea/ coffee	Water	-	missing
15. Legumes (e.g. beans, chickpeas, lentils)	How many servings of each of the following foods do you eat per week?	Qual:	0	≤ 1	2-3	3-4	5-6	≥ 7	-		
		0-4	0 pts	1 pts	2 pts	3 pts	4 pts	0 pts			
		FFQ:	<1.5		-	1.5≤x<3	3≤x<6.5	≥ 6.5	missing		
16. Nuts (e.g. walnuts, almonds, hazelnuts, pistachio, peanuts)	How many servings of each of the following foods do you eat per week?	Qual:	0	≤ 1	2-3	3-4	5-6	≥ 7	-		
		0-4	0 pts	1 pts	2 pts	3 pts	4 pts	0 pts			
		FFQ:	<1.5		-	1.5≤x3	3≤x<6.5	≥ 6.5	missing		
fh. Meals eaten at firehouse	How many meals per week do you usually eat at the firehouse (or on work time)?	Qual	≤ 1	2-4	5-7	8-10	11-13	14 or more	missing		
		weight	1/21	3/21	6/21	9/21	12/21	17/21	0.5		

FFQ: Harvard 131-item semiquantitative food frequency questionnaire, fh: proportion of meals consumed at the firehouse, mMDS: modified Mediterranean Diet Score, pts: points, Qual: Qualtrics lifestyle questionnaire.
[a]Weighted by the proportion of meals at home relative to the total number of meals per week (breakfast + lunch + dinner)
[b]Weighted by the proportion of meals at the firehouse relative to the total number of meals per week (breakfast + lunch + dinner)

© 2023 Hershey MS et al. *JAMA Network Open*.

eFigure 1. Influence Analysis Shows the Influence of Each Imputation Model Estimate on the Overall Effect Size Estimates for A) 6-Month and B) 12-Month Between-Group Differences in mMDS Changes

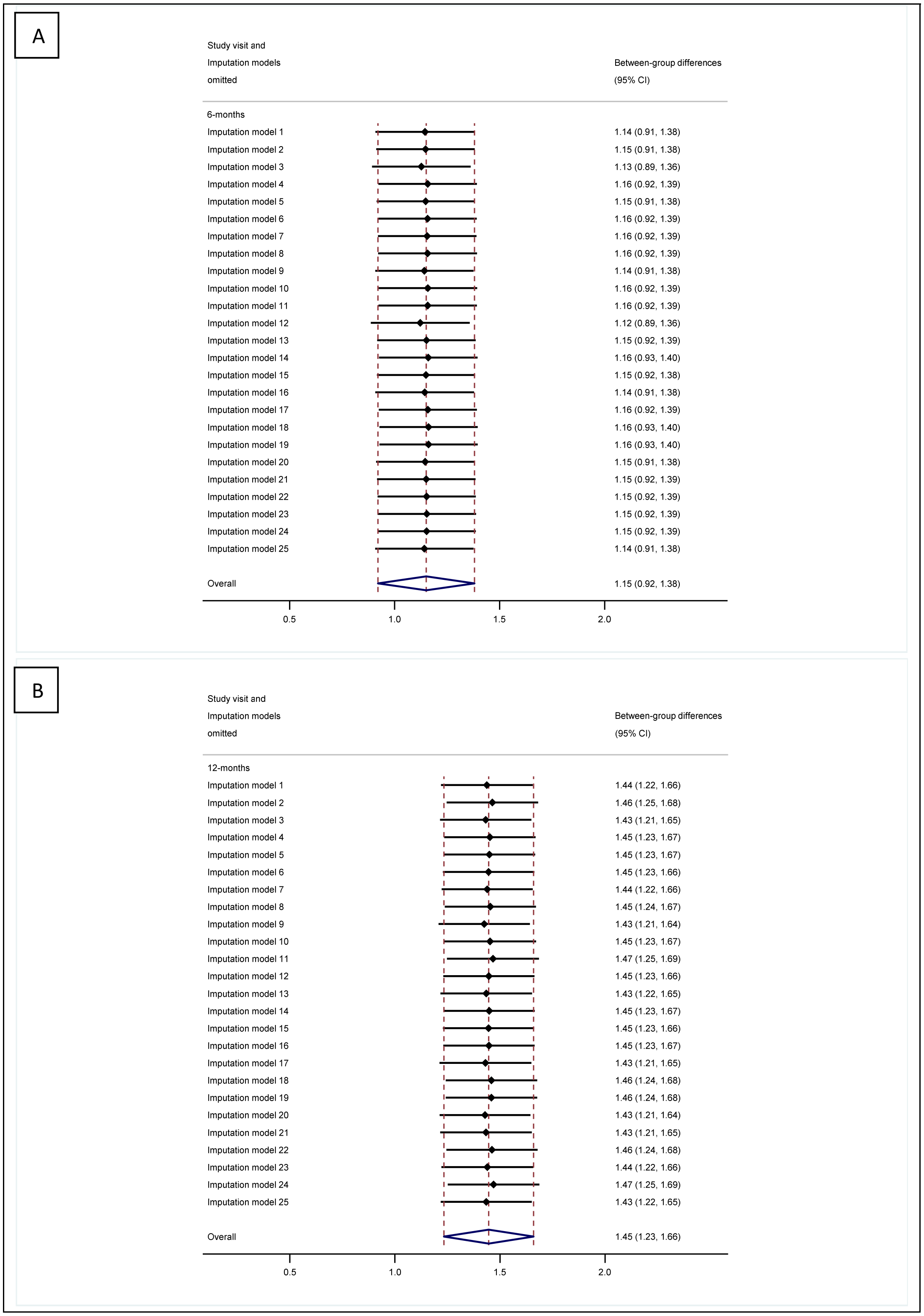

CI: confidence interval

An estimate is suspected of excessive influence if the point estimate of its "omitted" analysis lies outside the 95%CI of the "overall" analysis or if the omitted estimate differs in significance relative to the "overall" analysis.

© 2023 Hershey MS et al. *JAMA Network Open*.

eFigure 2. Quality Check for Multilevel Multiple Imputation Model #1; Histograms Show a Modified Mediterranean Diet Score (0-51 Points) for the Completed vs Observed Datasets and Mean (SD) by Study Group

Original observations for mMDS scores

Completed dataset includes original and imputed mMDS

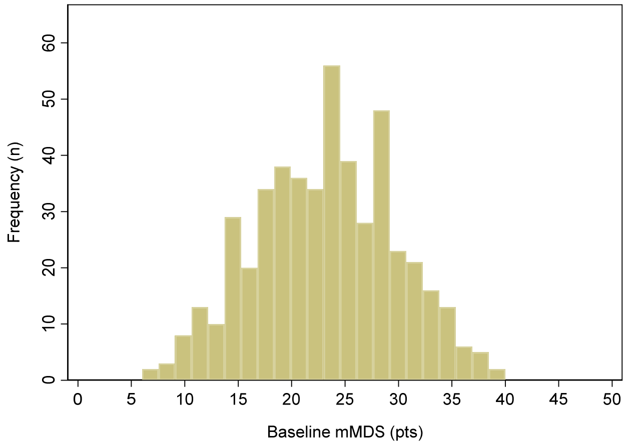

Control (n=244): mean=23.41 (SD: 6.34)
MED (n=240): mean=22.87 (SD: 6.78)

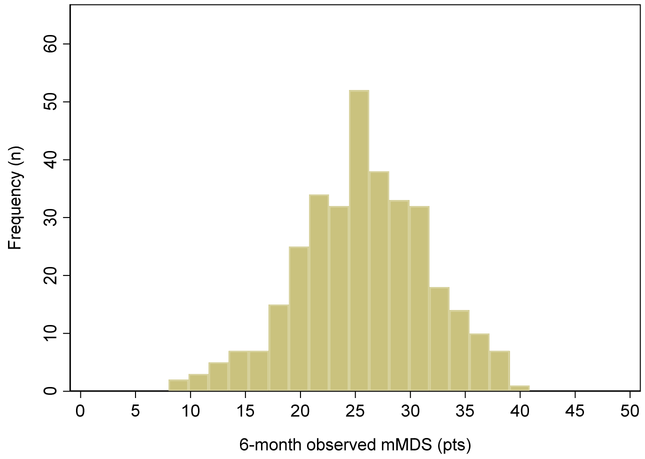

Control (n=184): mean=25.26 (SD: 5.73)
MED (n=151): mean=26.20 (SD: 6.15)

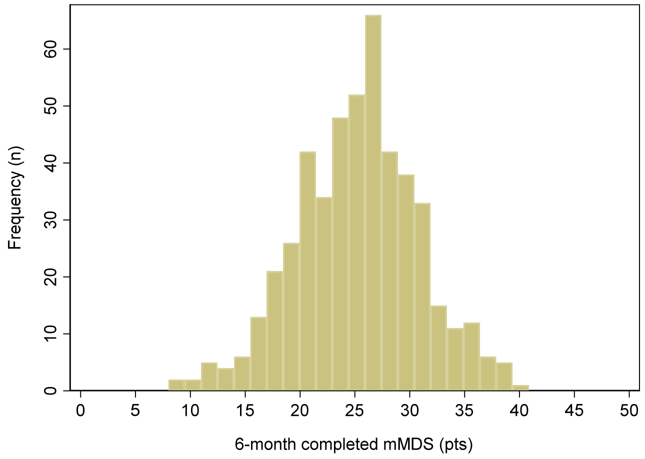

Control (n-244): mean= 24.92 (SD: 5.42)
MED (n=240): mean=25.58 (SD: 5.62)

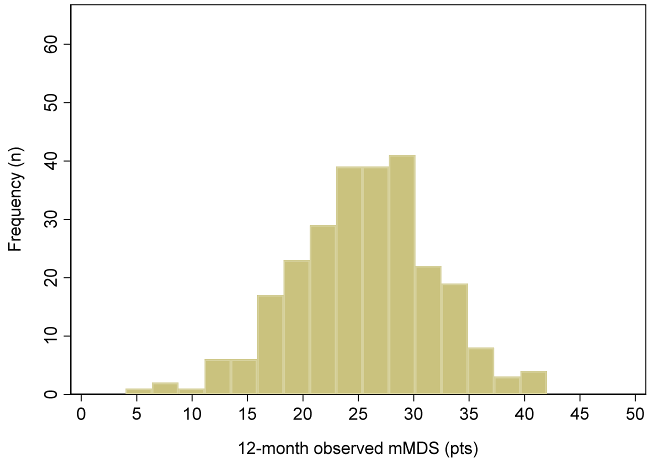

Control (n=160): mean=24.79 (SD: 6.14)
MED (n=100): mean=26.68 (SD: 6.42)

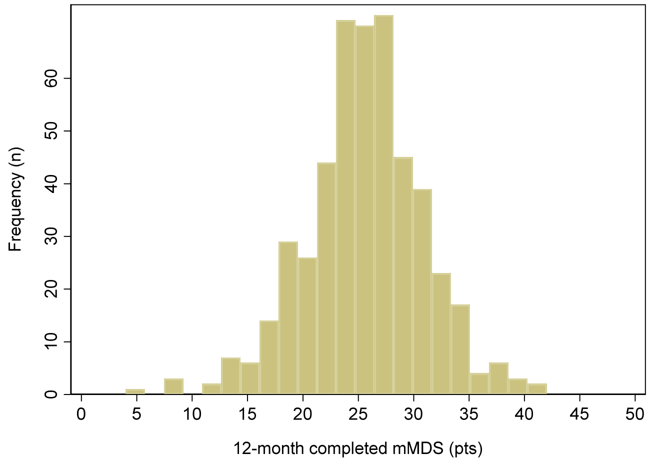

Control (n=244): mean= 24.99 (SD: 5.48)
MED (n=240): mean= 26.17 (SD: 5.15)

© 2023 Hershey MS et al. *JAMA Network Open*.

eFigure 3. Quality Check for Multilevel Multiple Imputation Model #1; Kernel Density Plots Show Observed, Completed, and Imputed Modified Mediterranean Diet Score Values for 6- and 12-Month Follow-Up

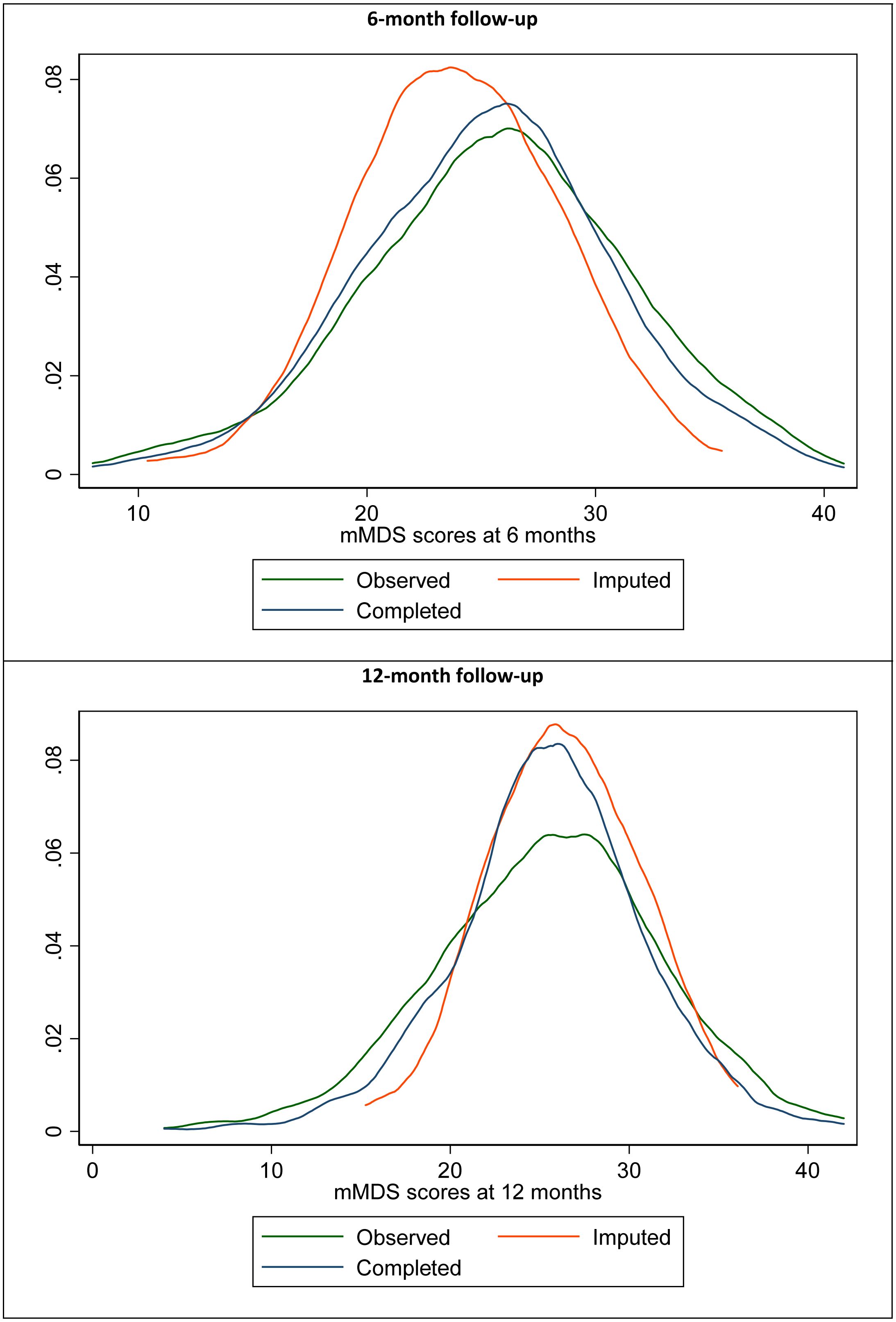

© 2023 Hershey MS et al. *JAMA Network Open*.

eTable 6. Quality Check for Multilevel Multiple Imputation Model #1; Summary Statistics Compare Observations With Completed Dataset Values for the Modified Mediterranean Diet Score at 6- and 12-Month Follow-Up

	N	Mean	Std. Dev.	min	max
**mMDS scores**					
mMDS	484	23.143	6.564	6	40
mMDS0m	484	23.143	6.564	6	40
mMDS 6m	335	25.687	5.933	8	40.857
mMDS6m	484	25.246	5.524	8	40.857
mMDS 12m	260	25.516	6.307	4	42
mMDS12m	484	25.579	5.349	4	42
**6-month mMDS items**					
mMDS6m_1	335	2.707	0.788	0	4
mMDS_6m1	484	2.612	0.865	0	4
mMDS6m_2	335	1.699	0.767	0	4
mMDS_6m2	484	1.696	0.804	0	4
mMDS6m_3	335	1.919	0.802	0	4
mMDS_6m3	484	1.921	0.810	0	4
mMDS6m_4	335	2.815	1.197	0	4
mMDSc_6m4	484	2.731	1.235	0	4
mMDS6m_5	335	3.412	1.882	0	5
mMDS_6m5	484	3.335	1.895	0	5
mMDS6m_6	335	2.639	1.806	0	4
mMDS_6m6	484	2.543	1.814	0	4
mMDS6m_7	335	2.594	1.802	0	4
mMDS_6m7	484	2.624	1.780	0	4
mMDS6m_8	335	3.287	1.726	0	5
mMDS_6m8	484	3.124	1.773	0	5
mMDS6m_9	335	2.155	1.880	0	4
mMDS_6m9	484	2.031	1.865	0	4
mMDS6m_10	335	2.91	1.702	0	4
mMDS_6m10	484	2.839	1.714	0	4
mMDS6m_11	335	2.552	0.917	0	4
mMDS_6m11	484	2.483	0.942	0	4
mMDS6m_12	335	1.122	0.793	0	4
mMDS_6m12	484	1.153	0.812	0	4
mMDS6m_13	335	0.257	0.670	0	2
mMDS_6m13	484	0.277	0.691	0	2
mMDS6m_14	335	2.14	1.477	0	4
mMDS_6m14	484	2.128	1.484	0	4
mMDS6m_15	335	0.746	0.885	0	4
mMDS_6m15	484	0.779	0.947	0	4
mMDS6m_16	335	1.137	1.173	0	4
mMDS_6m16	484	1.081	1.156	0	4
fhh6m	335	0.275	0.107	0.048	.833
impfhh_6m	484	0.272	0.089	0.048	.833
**12-month mMDS items**					
mMDS12m_1	260	2.785	0.800	1	4
mMDS_12m1	484	2.705	0.815	0	4
mMDS12m_2	260	1.681	0.802	0	4
mMDS_12m2	484	1.676	0.761	0	4
mMDS12m_3	260	2.004	0.808	0	4
mMDS_12m3	484	1.959	0.815	0	4
mMDS12m_4	260	2.977	1.083	0	4

© 2023 Hershey MS et al. *JAMA Network Open*.

mMDS_12m4	484	2.996	1.101	0	4
mMDS12m_5	260	3.223	1.964	0	5
mMDS_12m5	484	3.246	1.910	0	5
mMDS12m_6	260	2.565	1.805	0	4
mMDS_12m6	484	2.806	1.683	0	4
mMDS12m_7	260	2.6	1.831	0	4
mMDS_12m7	484	2.469	1.852	0	4
mMDS12m_8	260	3.054	1.821	0	5
mMDS_12m8	484	3.043	1.800	0	5
mMDS12m_9	260	2.25	1.846	0	4
mMDS_12m9	484	2.31	1.829	0	4
mMDS12m_10	260	2.712	1.796	0	4
mMDSo_12m10	484	2.647	1.811	0	4
mMDS12m_11	260	2.488	0.976	0	4
mMDS_12m11	484	2.471	0.951	0	4
mMDS12m_12	260	1.146	0.787	0	4
mMDS_12m12	484	1.209	0.826	0	4
mMDS12m_13	260	0.223	0.631	0	2
mMDS_12m13	484	0.252	0.664	0	2
mMDS12m_14	260	1.992	1.378	0	4
mMDS_12m14	484	2.008	1.397	0	4
mMDS12m_15	260	0.727	0.891	0	4
mMDS_12m15	484	0.711	0.891	0	4
mMDS12m_16	260	1.192	1.196	0	4
mMDS_12m16	484	1.198	1.223	0	4
fhh12m	260	0.258	0.115	0.048	.833
impfhh_12m	484	0.251	0.084	0.048	.833
**cardiometabolic parameters**					
BMI0m	470	30.033	4.413	19.515	51.563
BMI_0m	484	30.049	4.372	19.515	51.563
BMI6m	336	30.004	4.594	15.334	55.099
BMI_6m	484	29.936	4.227	15.334	55.099
BMI12m	263	29.954	4.529	20.539	53.125
BMI_12m	484	29.706	4.042	20.539	53.125
bodyfat0m	468	28.143	6.525	8	50.4
bodyfat_0m	484	28.115	6.530	8	50.4
bodyfat6m	336	27.993	6.579	12.9	55.9
bodyfat_6m	484	28.142	6.154	12.9	55.9
bodyfat12m	263	27.944	6.875	13.2	56.1
bodyfat_12m	484	27.882	6.170	13.2	56.1
waistcircumference0m	469	39.259	4.941	26.5	59
waistcircumference_0m	484	39.244	4.894	26.5	59
waistcircumference6m	336	38.629	4.886	27	57.5
waistcircumference_6m	484	38.555	4.659	27	57.5
waistcircumference12m	263	39.502	4.880	28	61
waistcircumference_12m	484	38.929	4.559	27.599	61
totalcholesterol0m	473	196.734	37.434	94	315
totalcholesterol_0m	484	196.733	37.111	94	315
totalcholesterol6m	276	195.924	38.296	106	407
totalcholesterol_6m	484	196.365	31.194	106	407
totalcholesterol12m	209	194.833	36.481	108	327
totalcholesterol_12m	484	195.853	27.200	108	327
hdlcholesterol0m	473	48.594	11.338	18	87
hdlcholesterol_0m	484	48.736	11.395	18	87

© 2023 Hershey MS et al. *JAMA Network Open*.

hdlcholesterol6m	276	48.543	12.622	14	101
hdlcholesterol_6m	484	48.999	12.454	14	101
hdlcholesterol12m	209	48.029	12.302	21	93
hdlcholesterol_12m	484	49.203	11.865	15.764	93
ldlcholesterol0m	465	123.187	32.428	41	230
ldlcholesterol_0m	484	123.187	32.315	41	230
ldlcholesterol6m	271	121.579	31.110	51	216
ldlcholesterol_6m	484	121.175	28.116	-122.097	216
ldlcholesterol12m	206	122.204	31.841	30	247
ldlcholesterol_12m	484	122.547	24.045	30	247
glucose0m	473	99.465	19.408	45	272
glucose_0m	484	99.143	19.755	45	272
glucose6m	293	100.369	26.916	43	402
glucose_6m	484	100.094	27.205	33.606	402
glucose12m	213	99.939	25.027	61	318
glucose_12m	484	100.41	25.125	29.011	318
triglicerides0m	472	125.331	76.019	28	640
triglicerides_0m	484	125.629	78.628	-61.855	640
triglicerides6m	276	141.228	244.861	31	3874
triglicerides_6m	484	136.456	194.550	-139.496	3874
triglicerides12m	209	126.885	91.819	31	845
triglicerides_12m	484	121.469	89.352	-111.549	845
**covariates**					
age0m	470	45.709	8.152	26	68
age_0m	484	45.709	8.033	26	68
calories0m	424	2407.4	1116.785	0	7559.29
calories_0m	484	2407.4	1045.120	0	7559.29
tobacco_status0m	322	0.677	0.897	0	2
tobacco_status_0m	484	1.45	0.798	0	2
physical_activity0m	314	1.532	0.711	0	2
physical_activity_0m	484	2.529	0.709	1	3
chronicdisease0m	480	0.30	0.459	0	1
chronicdisease_0m	484	1.298	0.458	0	1
sex0m	484	.056	0.230	0	1
sex_0m	484	1.056	0.230	1	2
race0m	484	1.2	0.467	1	3
race_0m	484	1.2	0.467	1	3
clusters0m	484	23.959	15.297	1	52
clusters_0m	484	23.959	15.297	1	52
group0m	484	1.496	0.501	1	2
group_0m	484	1.496	0.501	1	2

Original observations in *Feeding America's Bravest* are followed by the completed dataset values (observed + imputed) from imputation model #1 in the gray-highlighted rows. For the 16 components of the mMDS, variables are labeled with a suffix that indicates study visit (0m: baseline, 6m: 6-month, and 12m: 12 month) and the number of the mMDS component (1-16), which correspond to the mMDS components presented in eTable 5.

© 2023 Hershey MS et al. *JAMA Network Open*.

eMethods. Original Study Design and Intervention

Study design

PHASE I (first 12 months)

- *MED*: Mediterranean Diet Nutritional Intervention for 12 months.
- *Control*: No intervention, instructed to follow their usual diet.

PHASE II (last 12 months)

- *MED*: "Self-sustained continuation" - participants were encouraged to maintain eating Mediterranean style on their own using the support of the discount program, the study website and the support of fellow firefighters and family for the last 12 months.
- *Control*: Mediterranean Diet Nutritional Intervention for 6 months and self-directed maintenance diet intervention for the last 6 months. After the first year, for ethical reasons of offering a similar opportunity to receive potential health benefits, the control group received a 6-month MED as part of their incentives to encourage participation in the trial from the outset.

eFigure 4. Timeline of data collection (2016-2019).
Study visits are marked by the first corresponding date of data received or retrieved.

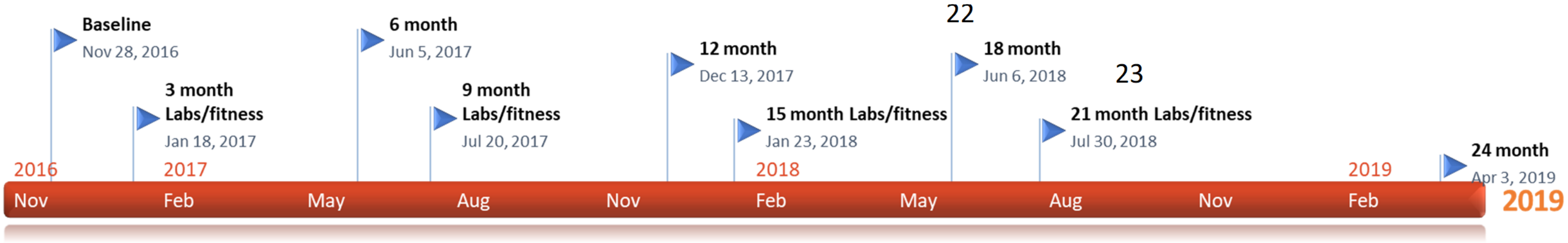

The Mediterranean Diet Nutritional Intervention (MED)

The present intervention incorporated behavior change components to promote the Mediterranean Diet, including nutrition knowledge and attitudes, environmental change, social context, and fiscal incentives. The extent to which consented participants personally engaged in MED was voluntary, and the additional time commitment required for the study was nominal. MED included educational materials; opportunities for discounted access to healthy foods consistent with the Mediterranean diet (e.g., extra-virgin olive oil, nuts, fruits, vegetables, legumes, whole-unrefined grains, and lean proteins) for both participating firefighters and their families; peer education/support, on-line learning, and email encouragement and reminders. Newly created intervention resources included a firefighters' Mediterranean food pyramid, grocery shopping list, "on the go" eating guide, fire station cooking demonstrations, "firefighter favorites recipes", dietitian-led video guides, and video interviews with exemplary firefighters following the Mediterranean diet. To address the most important barriers identified by the firefighters in a focus group previous to the intervention, time and cost, a discount program was established with Kroger

© 2023 Hershey MS et al. *JAMA Network Open*.

supermarkets for select foods, such as fruits, vegetables, olive oil, poultry, and seafood. Each of these aspects of the intervention is explained in further detail below.

Specific topics that were addressed in the group educational sessions, the videos, and/or the chef demonstrations included:

1. Basic Mediterranean Diet principles;
2. The benefits of Olive oil and nuts and how to introduce them into the diet;
3. Overcoming Challenges while following a Mediterranean Diet;
4. How to plan a weekly menu and shopping list;
5. How to choose wisely when eating out (restaurants/parties);
6. Making smart choices regarding "Fast food".

<u>Web-based</u> (ongoing throughout entire study period)

- Study website and study blog with online resources available to firefighters in the intervention group 24/7. https://www.hsph.harvard.edu/firefighters-study/feeding-americas-bravest/
- Email reminders were sent periodically to remind firefighters receiving the intervention how to access online study materials as well as announce new resources and upcoming events like educational sessions with dietitian and chef demonstrations.

<u>Small group educational sessions</u> (periodic)

The initial and periodic small group (5-20 firefighters) sessions were led by the local Public Safety Medical (PSM) team. Through focus groups before the onset of the intervention, firefighters expressed a preference for short, hands-on learning sessions and videos rather than intensive, classroom-style learning.

- <u>Cooking Demonstrations</u>:
  - Demonstration #1 was hosted at an IFD firehouse by Lt. Michael Cacciola. This demonstration was open to firefighters working in that particular firehouse; however, the demonstration was also filmed for the TV Show "FireHouse Kitchen".
  - Demonstration #2 was hosted at a Kroger grocery store cooking classroom by Chef Maria Loi. This demonstration was open to IFD and Fishers FD firefighters and their partners or spouses as well as the general public.
  - Demonstration #3 was hosted at an IFD firehouse by Chef Maria Loi. This demonstration was open to firefighters working in that particular firehouse.

- <u>Educational Sessions with a dietitian</u>:
  The educational sessions were led by Indiana-based dietitian and Health/Fitness Specialist, Heather Fink, who is contracted with PSM and has a long experience working with firefighters. The sessions included: didactic presentations, interactive discussions and cooking demonstrations that followed the Mediterranean diet recommendations (see below). The educational sessions were held in the evening at the local firefighter Union hall and were open to firefighters currently receiving the intervention and their partners or spouses. Overall, the educational sessions covered the 6 topics mentioned above.

© 2023 Hershey MS et al. *JAMA Network Open*.

eTable 7. MEDITERRANEAN DIET: GENERAL RECOMMENDATIONS

FOOD	GOAL
**RECOMMENDATION**	
1. Olive oil (extra virgin if possible)	Main culinary fat or ≥4 tbsp/day
2. Tree nuts and peanuts (peanut butter without added sugars or hydrogenated fat)	≥3 servings/wk
3. Fresh fruit	≥3 servings/day
4. Vegetables	≥2 servings/day
5. Fish (including fatty fish) and seafood	≥3 servings/wk
6. Legumes (chickpeas, beans, lentils)	≥3 servings/wk
7. Sofrito[a]	≥2 servings/wk
8. Fresh Herbs, Allium (onion, garlic, etc)	≥2 servings/day
9. Yogurt	≤2 servings/day
10. White meat	Instead of red meat (2-3 servings/wk)
11. Whole grains/cereals[b]	Instead of refined grains
12. Wine with meals (optional, only for habitual drinkers)[c]	≥7 glasses/wk
**DISCOURAGE**	
13. Soda drinks (encourage water as a beverage)	<1 drink/day
14. Spread fats	<1 serving/day
15. Red and processed meats	<1 serving/day (< 2 servings/wk)
16. Commercial bakery foods sweets, and pastries[d]	<3 servings/wk
17. Fast food	≤1/wk

[a] Sofrito is a tomato-sauce made with onion and garlic, slowly simmered with olive oil.
[b] Women 75 g/day, men 90 g/day or ≥5 servings/day. Whole grains brown rice, whole grain bread and pasta.
[c] Optional and only for habitual drinkers without alcohol-related health problems.
[d] Homemade sweets are preferable to store-bought or commercially produced sweets.

- Educational Videos:
  A series of educational videos were filmed featuring Heather Fink that covered many of the 6 topics listed above. The videos were posted on the study intervention website and promoted to firefighters in the intervention group by email and during study follow-up visits.

Peer-education and support

The Research Team formed a study Advisory Board at IFD made up of study participants and fire service leaders in health and safety, peer support, and logistics. The Advisory Board reviewed study intervention materials, provided feedback to investigators, helped plan study events, and offered guidance as needed. Firefighters also had access to several video testimonials on the study website, where other firefighters described their successes with the Mediterranean Diet.

Kroger Grocery Coupons (ongoing throughout entire study period)

- Kroger provided a series of paper coupons for grocery items consistent with a Mediterranean diet.
- The coupons were distributed quarterly (packet of coupons valid for the next 3 months) to intervention firehouses by mail. Each coupon delivery included a letter from the study team and several recipes from the study intervention website that aligned with the coupons.

© 2023 Hershey MS et al. *JAMA Network Open*.

- Extra coupons were also made available to firefighters in the intervention group during study follow-up visits, which occurred every 6 months.
- Firefighters were encouraged to use the coupons both at the firehouse and at home with their families.

<u>Mediterranean Diet Food Samples</u> (periodic)

- Firefighters receiving the intervention received several deliveries of free Mediterranean diet foods, including extra virgin olive oil, high protein pasta, olives, and almonds, to their firehouse.
- The food deliveries included a letter from the study team and several recipes from the study intervention website.

<u>Other</u> (periodic)

- During study follow-ups every 6 months, firefighters in the intervention group received extra Kroger grocery coupons, copies of printed materials and recipes from the study website, and flyers with instructions for how to access the study website.

<u>Firefighter Mediterranean Diet Food Pyramid Poster</u>:

- Firehouses receiving the intervention received a printed firefighter Mediterranean diet food pyramid poster to display in the firehouse and magnets in their homes.

eFigure 5. Firefighter Mediterranean Diet Food Pyramid.

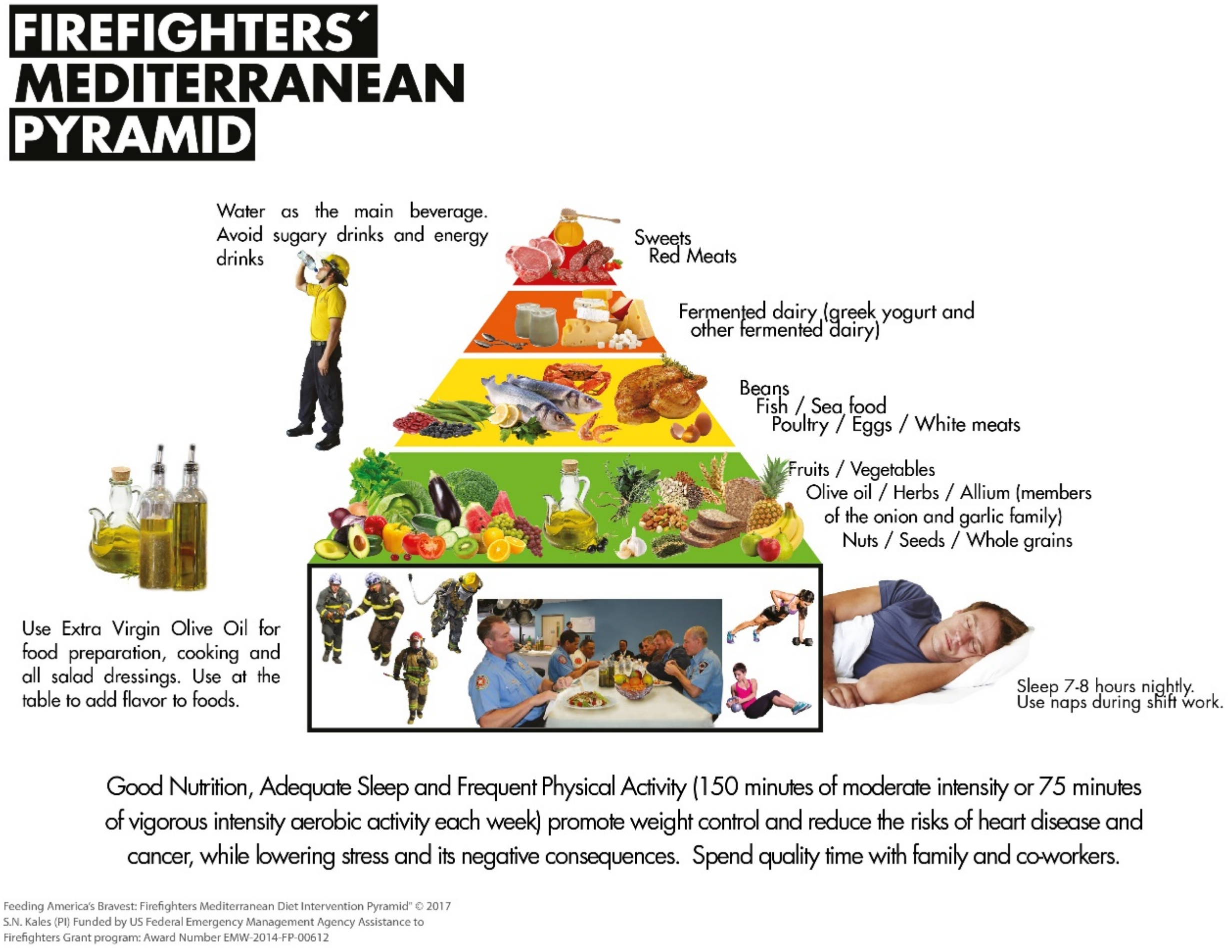

© 2023 Hershey MS et al. *JAMA Network Open*.
