# Supplementary material for: Effect of a Nutrition Intervention on Mediterranean Diet Adherence Among Firefighters: A Cluster Randomized Clinical Trial
Source: JAMA Netw Open. 2023 Aug 17;6(8):e2329147. doi: 10.1001/jamanetworkopen.2023.29147 (PMC10436136; doi:10.1001/jamanetworkopen.2023.29147)
Supplement: Supplement 3. — Data Sharing Statement [file jamanetwopen-e2329147-s003.pdf]

Data Sharing Statement

Hershey. Effect of a Nutrition Intervention on Mediterranean Diet Adherence Among Firefighters. *JAMA Netw Open*. Published August 17, 2023. doi:10.1001/jamanetworkopen.2023.29147

Data
Data available: No

Additional Information
Explanation for why data not available: The study protocol and all data, including individual participant data, data dictionary that defines each field of the data set, and supporting documentation (statistical/analytic code) are available upon request.
